# *In silico* profiling of systemic effects of drugs to predict unexpected interactions

**DOI:** 10.1038/s41598-018-19614-5

**Published:** 2018-01-25

**Authors:** Sunyong Yoo, Kyungrin Noh, Moonshik Shin, Junseok Park, Kwang-Hyung Lee, Hojung Nam, Doheon Lee

**Affiliations:** 1 Department of Bio and Brain Engineering, Korea Advanced Institute of Science and Technology (KAIST), Daejeon, 34141 Republic of Korea; 2 Bio-Synergy Research Center, Daejeon, 34141 Republic of Korea; 3 School of Electrical Engineering and Computer Science, Gwangju Institute of Science and Technology (GIST), Gwangju, 61005 Republic of Korea

## Abstract

Identifying unexpected drug interactions is an essential step in drug development. Most studies focus on predicting whether a drug pair interacts or is effective on a certain disease without considering the mechanism of action (MoA). Here, we introduce a novel method to infer effects and interactions of drug pairs with MoA based on the profiling of systemic effects of drugs. By investigating propagated drug effects from the molecular and phenotypic networks, we constructed profiles of 5,441 approved and investigational drugs for 3,833 phenotypes. Our analysis indicates that highly connected phenotypes between drug profiles represent the potential effects of drug pairs and the drug pairs with strong potential effects are more likely to interact. When applied to drug interactions with verified effects, both therapeutic and adverse effects have been successfully identified with high specificity and sensitivity. Finally, tracing drug interactions in molecular and phenotypic networks allows us to understand the MoA.

## Introduction

Most complex diseases are caused by a complicated interplay of various biological processes and dysfunctional systems. However, traditional drug discovery paradigm, ‘the single drug, single target’, has limitations in many aspects of the complex disease treatment. Single drug acting on a single target in a disease system may ignore complex interactions between drugs and their target proteins^1^. Compared to single drug treatments, it is now evident that drug combinations have a number of advantages such as increase of therapeutic effects, reduction of drug dosage and decrease of toxicity and side effects^2^. However, unexpected adverse effects may also occur due to drug-drug interactions (DDIs) resulting from various processes^3^. On this account, significant attention to overall phenotypic effects of drug interactions is necessary to discover drug combinations with increased therapeutic effects and reduced adverse effects. While there is a relatively large number of *in vitro* and *in silico* methods identifying phenotypic effects of a single drug^4,5^, only a few methods attempt to investigate phenotypic effects of drug interactions^6,7^.

Systematic *in vitro* approaches have been used to identify effective drug combinations by using a high-throughput screening technique^8–10^. However, large-scale experiments are needed for each possible drug combination, which increases cost and time exponentially with the increase of the number of drugs. Therefore, systematic *in silico* approaches have been proposed to investigate potential drug combinations by calculating drug similarity based on various types of drug information, including chemical structure, target proteins, ATC code and side effects^11,12^. Although these methods have good performance in predicting drug combinations, they require various types of annotated data and cannot predict phenotypic effects of drug combinations. Alternatively, some computational approaches have been developed to predict potential drug combinations based on network-based analysis from drug-induced gene expression profiles^13–15^. These methods construct a backbone network, e.g., functional, protein interaction or drug-target interactions, and identify drug sets with similar response on the network. While these methods can estimate whether a drug combination is effective on a certain disease, it is difficult to predict the overall effects on the human body, since a large amount of gene expression data is required to consider many phenotypes. At the same time, several DDI prediction methods, such as similarity-based, knowledge-based or mechanism-based methods, have been proposed^16–18^. Most of these methods only focus on predicting DDIs in given drug pairs without showing their potential phenotypic effects. Only a few knowledge-based approaches predicted adverse effects of drugs and DDIs based on adverse drug reaction (ADR) reports and Electronic Health Records (EHRs)^6,7^. However, these methods suffer from several limitations in the use of spontaneous ADR reporting system, including sampling variance and reporting biases^6,19,20^. More importantly, they seldom consider the complicated mechanism of action (MoA) of drug effects and interactions in biological systems.

Here, we present a phenotype-based *in silico* method to predict effects and interactions of drug pairs based on the profiling of systemic effects of drugs. Recent studies have demonstrated that phenotypic effects of drugs can be utilized in predicting drug interactions by using them to calculate the similarity between drug pairs or as a core feature in machine learning^11,21–23^. We, therefore, hypothesized that drug pairs having similar phenotypic effects are more likely to interact with each other. To test this underlying hypothesis, we first generated profiles of systemic effects for each drug by investigating the propagated drug effects from a molecular network and mapped these results to a phenotypic network. Next, to identify the effects and interactions of drug pairs, the connectivity and closeness between phenotypes of drug profiles were calculated in the phenotypic network. By introducing systematic analysis on molecular and phenotypic networks, effects and interactions of drug pairs were identified together with the understanding of the underlying MoA. To investigate the coverage of our prediction, we applied our method to two sources for predicting both therapeutic indications and adverse effects of drug interactions. We found that predicted effects and interactions of drug pairs cover the large amounts of results which were reported in previous work.

To conclude, the novelty of our method is threefold: (i) to our knowledge, it is the first unbiased, phenotype-based *in silico* method which predicts drug effects and their interaction potential with quantitative assessment; (ii) by investigating systemic effects of drugs and their associations in molecular and phenotypic networks, it enables us to understand the MoA of drug interactions; and (iii) as a preliminary tool, our method can screen candidate drug combinations and notify us hazardous drug interactions.

## Results

### *In silico* analysis for identifying drug interactions and their phenotypic effects

**Figure 1:**
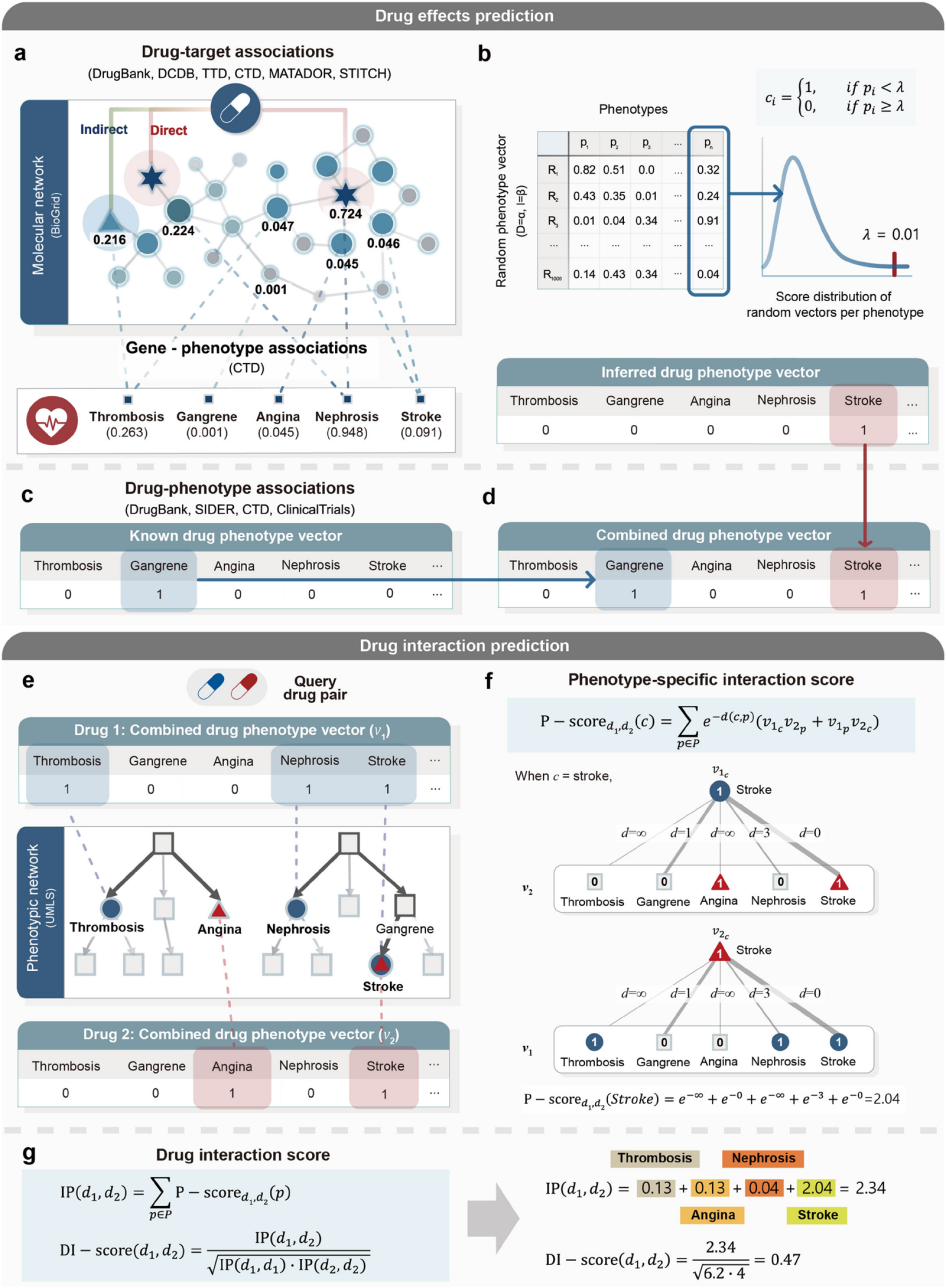
A systematic overview of the drug effect prediction. (**a**) Phenotype values of a drug were obtained by calculating propagated drug effects on the molecular network. In the molecular network, RWR algorithm was performed based on direct targets (star) and indirect targets (triangle), in which RWR results are shown as colored nodes. Based on gene-phenotype associations (dashed line), sums of gene values are mapped to phenotypes (square). (**b**) Effective phenotypes were filtered by comparing with random DPVs. Random DPVs of a drug were generated by randomly selecting targets while fixing the number of direct (*α*) and indirect (*β*) target proteins. For each drug, 1,000 random DPVs were generated, and phenotypes (*c*_*i*_) with an empirical *p*-value lower than 0.01 were selected to construct an inferred DPV. (**c**) A known DPV was constructed based on public databases. (**d**) A combined DPV was created by integrating known and inferred DPVs. (**e**) For a query drug pair, all phenotype values of each combined DPV were mapped to the phenotypic network. (**f**) The P-score of a phenotype was calculated by considering phenotype pairs containing this specific phenotype among the whole pairs with the closeness. Closeness was calculated by the distance between phenotype pairs of each combined DPV in the phenotypic network. (**g**) Interaction potential of a drug pair was quantified by the sum of the P-scores. The DI-score was calculated by normalizing interaction potential values with the geometric mean of the values obtained by calculating each interaction potential against itself.

We designed a novel algorithm to predict phenotypic effects of drug pairs and their interaction potential by investigating systemic effects of drugs from molecular to phenotypic networks. For a query drug pair, the algorithm works in five steps (Fig. 1): (i) constructing an inferred drug phenotype vector (DPV) by calculating systemic effects of a drug on the molecular network and filtering effective phenotype values; (ii) constructing a known DPV based on published databases; (iii) creating a combined DPV based on known and inferred DPVs; (iv) mapping phenotypes of the combined DPV on the phenotypic network; and (v) calculating phenotype-specific interaction scores (P-scores) and drug interaction scores (DI-scores) for a query drug pair based on their combined DPVs.

We generated DPVs which cover systemic effects, including both known and unexpected phenotypic effects, of individual drugs. Each DPV contains drug effects for 3,833 phenotypes defined by Medical Subject Headings (MeSH) and Online Mendelian Inheritance in Man (OMIM). For this, the inferred DPV was generated in three steps. In the first step, propagated drug effects were calculated by using the random walk with restart (RWR) algorithm on the molecular network (Fig. 1a). The effects of drugs are not limited to their direct targets, but they are further propagated to interacting proteins. Therefore, initial values of the molecular network were assigned to the drug targets, and their propagated effects were calculated by applying the RWR algorithm. In the second step, phenotype values were calculated by combining propagated drug effects based on gene-phenotype associations. Accordingly, phenotypes have high values when a drug directly binds to phenotype associated genes or when drug targets are closely located to phenotype associated genes. In the third step, an inferred DPV was constructed by filtering effective phenotypes from the inferred list of phenotypes, which were calculated from the second step (Fig. 1b). When there were a large number of drug targets or phenotype associated genes, the phenotype tended to get a high value. The result was caused by the imbalance of the prior knowledge of drug targets, protein interactions and phenotype associated genes. To show that prior knowledge has high variation, we calculated the coefficient of variation (CV) which is defined as the ratio of the standard deviation to the mean. The CV of genes per phenotype (CV = 3.58), node degree of the molecular network (CV = 2.68) and direct and indirect targets per drug (CV = 3.94 and 6.80, respectively) were considered to be high variances since those CV values were higher than one^24^. To overcome this problem, we filtered effective phenotype values by comparing phenotype values with the randomly generated DPVs. The phenotype values were converted into Boolean values (one or zero) based on *p*-values, which were calculated from the distribution of phenotype values of random DPVs. The list of phenotype values of a drug, determined by combining the molecular and phenotypic networks, was defined as the inferred DPV. The average number of phenotypes per inferred DPV was 116.1. Next, we constructed the known DPV by collecting drug-phenotype associations from published databases (Fig. 1c). The average number of phenotypes per known DPV was 57.4. Finally, a combined DPV was generated by integrating both the known and inferred DPVs, which has a large coverage of phenotypic effects including verified and unexpected drug-phenotype associations (Fig. 1d). Through this process, the average number of phenotypes were increased (27.4%) for 2,434 drugs among a total of 5,441 drugs. Consequently, we generated combined DPVs for 5,441 approved and investigational drugs, which contain an average of 160.8 phenotypes. The distribution of the number of targets associated with drugs, genes associated with phenotypes and phenotypes associated with drugs is provided in Supplementary Fig. 1.

To investigate the relationship between drugs, we mapped phenotype values of combined DPVs on the phenotypic network (Fig. 1e). In the phenotypic network, phenotype nodes were connected when they shared some common properties or were related by definition^25^. Therefore, similar phenotypes were closely linked with shared properties. The average distance of the random phenotype pair was 6.5 and the average distance of the phenotype pair of the known drug combination was 3.4 in the phenotypic network. This indicates that the drugs are more likely to have similar phenotypic effects when they interact with each other. For a given query drug pair, all phenotype terms in both combined DPVs were considered as candidate phenotypic effects of the drug pair. To filter out meaningless phenotypes, all candidates were ranked by P-scores, which were calculated by considering connectivity and closeness between phenotype pairs containing this specific phenotype among the whole pairs (Fig. 1f). To further predict whether the drug pair interacts, the interaction potential was quantified by aggregating all P-scores of the drug pair (Fig. 1g). If a drug pair shares a relatively higher number of similar phenotypes, the drug pair will have high scored phenotype terms, increasing the interaction potential. Finally, we calculated DI-scores which normalize the interaction potential by dividing it by the geometric mean of the values obtained by calculating each interaction potential against itself. We provide a web interface for all predicted drug interactions and their ranked phenotypic effects, which is available at http://biosoft.kaist.ac.kr/DPV.

### Systemic effects of drugs are predicted by network propagation from molecule to phenotype

In this study, the prediction of systemic effects of drugs on the molecular and phenotypic networks is an essential step to identify phenotypic effects of drug pairs and their interaction potential. Therefore, we first evaluated whether DPVs can be used to predict phenotypic effects of drugs by comparing them with randomized target DPVs. Inferred DPVs without known DVPs were used for a fair comparison (Fig. 1b). Also, randomized target DPVs were constructed by applying the same development strategies used for the inferred DPV, where only the targets were randomly selected while fixing the number of targets. Three types of phenotypic effect information, including therapeutic effects, side effects and potential candidate effects, were used as a gold standard, respectively (Table 1). We collected verified phenotypic effects from DrugBank indications for therapeutic effects, SIDER for side effects and CTD for potential candidate effects and used them as a gold standard positive set. To evaluate the prediction results, precision (*p*) and recall (*r*) values were calculated. Out of 4,990 therapeutic effects of 828 drugs, inferred DPVs covered 1,692 phenotypes (*p* = 0.033 and *r* = 0.339), which represents better performance compared to randomized target DPVs (*p* = 3.04 × 10^−4^ and *r* = 0.010). Similarly, for side effect prediction, inferred DPVs covered 7,570 phenotypes (*p* = 0.079 and *r* = 0.256) among the total 29,574 phenotypes of 643 drugs, and in potential candidate effect prediction, inferred DPVs covered 168,360 phenotypes (*p* = 0.381 and *r* = 0.557) among the total 302,514 phenotypes of 1,585 drugs. Overall performance represents that the prediction of phenotypic effects with inferred DPVs shows better performance compared to the prediction with randomized target DPVs.

**Table 1:** Precision and recall values of inferred DPV and randomized target DPV in predicting therapeutic effects, side effects, off-label effects and potential candidate effects.

	Inferred DPV		Randomized target DPV	
	Precision	Recall	Precision	Recall
Therapeutic effects	0.033 (±0.006)	0.339 (±0.024)	3.04 × 10^−4^ (±1.0 × 10^−6^)	0.010 (±1.0 × 10^−4^)
Side effects	0.079 (±0.003)	0.256 (±0.017)	2.92 × 10^−3^ (±1.0 × 10^−5^)	0.011 (±1.0 × 10^−4^)
Potential candidates	0.381 (±0.019)	0.557 (±0.064)	7.34 × 10^−3^ (±1.0 × 10^−5^)	9.65 × 10^−3^ (±1.0 × 10^−5^)

Next, we investigated the relationship between precision and recall according to *p*-value threshold. The precision and recall performance of the inferred DPVs are influenced by the *p*-value threshold in the process of calculating phenotype values (Fig. 1b). Setting a high *p*-value increases the number of phenotype candidates of the drug, which causes the precision to decrease but the recall value to increase. Conversely, setting a low *p*-value decreases the number of phenotype candidates, which causes the precision to increase but the recall value to decrease. The relationship between precision, recall and F1 scores according to *p*-value can be seen in the Supplementary Table 1.

Unlike our method, knowledge-based approaches, including OFFSIDES, predict drug effects from ADRs based on statistical analysis^6^. However, they suffer from several limitations in the use of spontaneous ADR reporting system, including sampling variance and reporting biases^6,19,20^. To demonstrate that our prediction is not biased to specific phenotype terms, we calculated phenotypic similarity for all drug pairs based on Jaccard index (JI). The Jaccard index of phenotypic effects between drugs is high when predictions are biased toward common phenotypic effects. From the result, average Jaccard index of inferred DPVs (JI = 2.2 × 10^−2^) was 2.95 times less than OFFSIDES (JI = 6.5 × 10^−2^). Overall, these findings indicate that inferred DPVs provide a high coverage and unbiased results in predicting phenotypic effects of drugs.

### Highly connected phenotypes represent potential phenotypic effects of drug pairs

**Figure 2:**
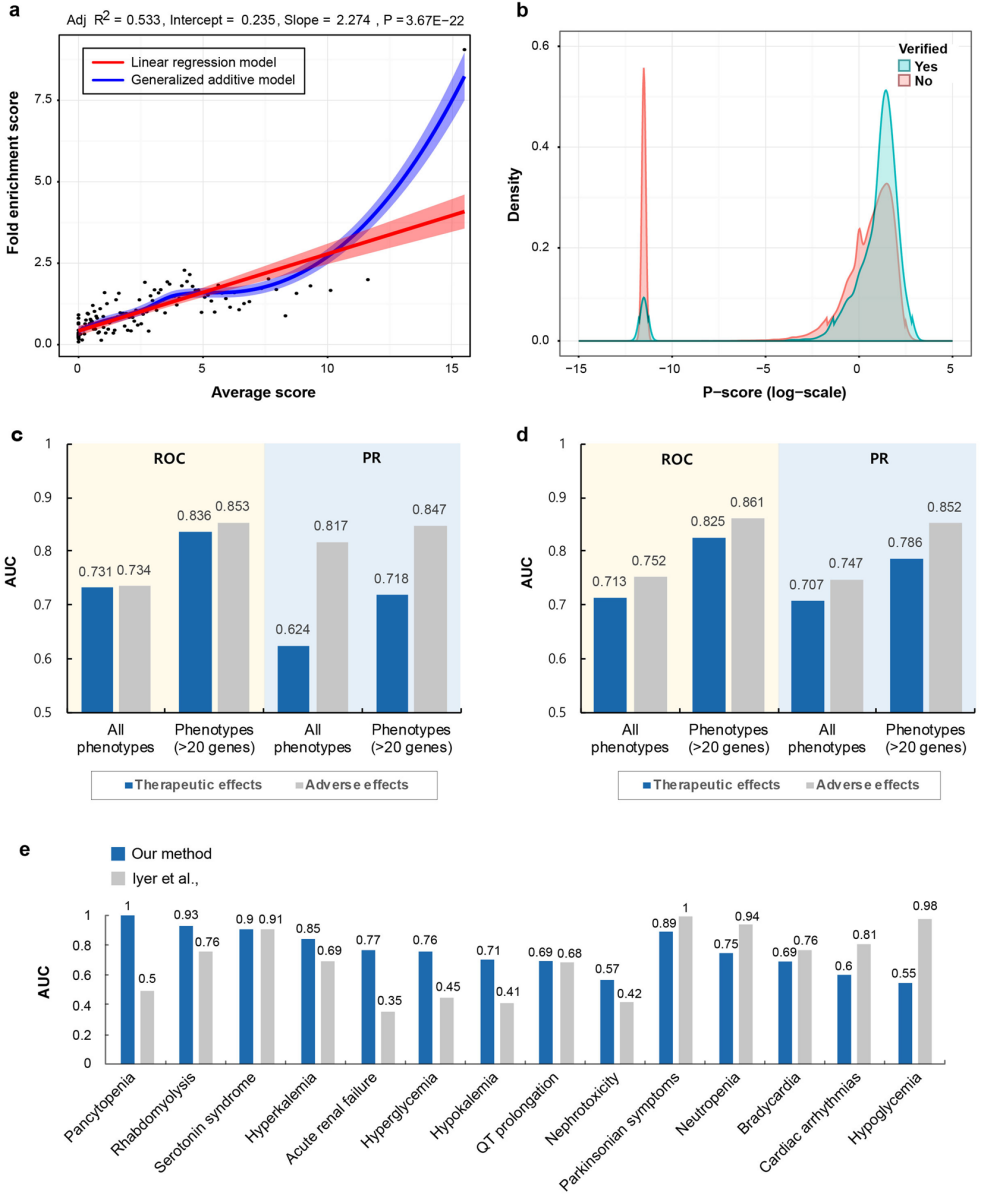
Performance evaluation in identifying phenotypic effects of drug interactions. (**a**) All possible phenotypes are ranked and binned into groups by their P-scores. FE of hits of known phenotypes in each bin (y-axis) is plotted against average P-score (x-axis). Linear regression model (red) and generalized additive model (blue) are used to fit the distribution of FE values. Red and blue outlines indicate 95% confidence interval. (**b**) The result of KDE: The P-score distributions show the relative separation between verified (blue) and unverified (red) phenotypic effects of drug combinations. (**c**,**d**) Average AUC scores of ROC and PR were calculated to evaluate the performance of phenotypic effects prediction. The known therapeutic and adverse effects were used as a gold standard positive set, respectively. Additionally, average AUC scores of ROC and PR were calculated for phenotypes with more than 20 related targets. Average AUC scores were calculated based on (**c**) drugs and (**d**) phenotypes, respectively. (**e**) Comparison of the AUROC between our method (blue) and Iyer method (gray). We used a gold standard built by Iyer study which contains 1,698 DDIs for 14 diseases.

Our method predicts phenotypic effects of drug pairs by calculating the connectivity and closeness between phenotypes of each drug, which are potential therapeutic or adverse effects. Using known therapeutic and adverse effects collected from DCDB^26^ and TWOSIDES^6^ as gold and silver standards respectively, we calculated the coverage of our predictions. We defined a set of candidate phenotypic effects of a drug pair as a union of phenotypes in combined DPVs. As a result, our predictions offered a large coverage in predicting therapeutic effects (63%) and adverse effects (41%) of drug pairs (Supplementary Fig. 2). However, for there were too many phenotype candidates, including false positives, we needed quantitative assessment to filter out meaningless phenotype candidates. To solve this problem, P-scores were calculated for all possible phenotypes in combined DPVs. Then we performed the fold-enrichment (FE) test to evaluate the correlation between the P-score and the likelihood of having known phenotypic effects. All candidate phenotypic effects were ranked by the P-score and binned into groups of 5,000 phenotypes. As a result, as P-scores increase, the number of known phenotypic effects has markedly increased, especially at top 10% region (Fig. 2a). We also performed kernel density estimation (KDE) to compare the density of distribution between verified and unverified phenotypic effect scores. Original distribution of verified and unverified phenotypic effect scores shows right-skewed distribution. Therefore, the log-transformed value of the P-score were used to better reveal the pattern of the data. As a result, the distribution of the verified phenotypic effect scores was more enriched on high score region than low score region, while the distribution of the unverified phenotypic effects showed opposite aspect (Fig. 2b). This indicates that verified phenotypic effects can be detected more readily if drug pairs have high P-scores. Also, the P-score shows a clear bimodal distribution, which can be used to filter out meaningless phenotypes. Next, average area under the curve (AUC) scores of receiver operating characteristic (ROC) and precision-recall (PR) curves for therapeutic effects of 1,093 drug combinations and adverse effects of 53,694 drug interactions were calculated respectively to examine the performance of the P-score (Fig. 2c). Additionally, in order to confirm whether the proposed method shows better performance when the amount of information is sufficient, 203 phenotypes with more than 20 related targets were selected from 3,833 phenotypes and average AUC scores of ROC and PR were calculated. As a result, we obtained the area under the receiver operating characteristic (AUROC) and area under the precision-recall curve (AUPR) scores for therapeutic effect (AUROC = 0.731 ± 0.021, AUPR = 0.624 ± 0.003) and adverse effect (AUROC = 0.734 ± 0.033, AUPR = 0.817 ± 0.015) predictions. In addition, when the genetic information associated with the phenotypes was sufficient, we were able to predict therapeutic (AUROC = 0.731 ± 0.021, AUPR = 0.624 ± 0.003) and adverse effects (AUROC = 0.734 ± 0.033, AUPR = 0.817 ± 0.015) with higher performance. Next, we calculated the ROC and PR performance for each phenotype and averaged them to normalize the occurrence of phenotypes (Fig. 2d). For this, we obtained phenotype ranking for each drug pair based on P-scores and then calculated the ROC and PR of the phenotype based on ranking in all drug pairs. This process was applied on all phenotypes, and the average values of AUROC and AUPR were calculated to normalize the phenotype occurrence. From the result, we confirmed that when phenotype occurrence is normalized, similar performance in predicting therapeutic (AUROC = 0.713 ± 0.032, AUPR = 0.707 ± 0.026) and adverse effects (AUROC = 0.752 ± 0.036, AUPR = 0.717 ± 0.024) is obtained. In addition, when genetic information related to the phenotype was sufficient, the therapeutic (AUROC = 0.825 ± 0.009, AUPR = 0.786 ± 0.008) and adverse effect (AUROC = 0.861 ± 0.012, AUPR = 0.852 ± 0.011) prediction performance was greatly increased. These results indicate that P-scores using combined DPVs are relevant for phenotypic effect prediction. Finally, we compared our predictions with Iyer study, one of the knowledge-based approaches predicting adverse effects of drug interactions^7^. The AUC scores of the adverse effect prediction of drug interactions were calculated by using TWOSIDES as a silver standard, yet there exists biases and errors toward ADR reports. To ameliorate this situation, we used a gold standard built by Iyer study which contains 1,692 DDIs for 14 diseases (Supplementary Data 1). Although this gold standard covers a relatively small number of drug interactions and diseases, we could evaluate the prediction performance in more strict condition. In comparison of the AUROC scores, our method showed better performance than Iyer study in predicting acute renal failure, hyperglycemia, hyperkalemia, hypokalemia, nephrotoxicity, pancytopenia, rhabdomyolysis and QT prolongation (Fig. 2e). Also, the lowest AUROC of our prediction result was 0.55 in hypoglycemia, while Iyer study did not perform well in predicting pancytopenia, acute renal failure, hyperglycemia, hypokalemia and nephrotoxicity, with AUROC scores less than or equal to 0.5. Moreover, our method has a definite advantage in offering insights on the MoA of drug interactions, which cannot be handled in knowledge-based approaches. Overall, our results indicate that the P-score can be used for identifying prioritized phenotypic effects of drug pairs.

### Drug pairs having similar phenotypes are more likely to interact with each other

To demonstrate whether the quantified phenotypic effects of drug pairs can be used to predict drug interactions, we first calculated DI-scores for all possible drug pairs among the list of FDA-approved and investigational drugs (Supplementary Data 2). We then evaluated the performance in predicting drug interactions which have therapeutic effects or adverse effects. As gold standard datasets, 1,093 drug combinations from DCDB^26^ and TTD^27^ were used for therapeutic effects and 29,074 DDIs from DrugBank^28^ and KEGG^29^ were used for adverse effects.

**Figure 3:**
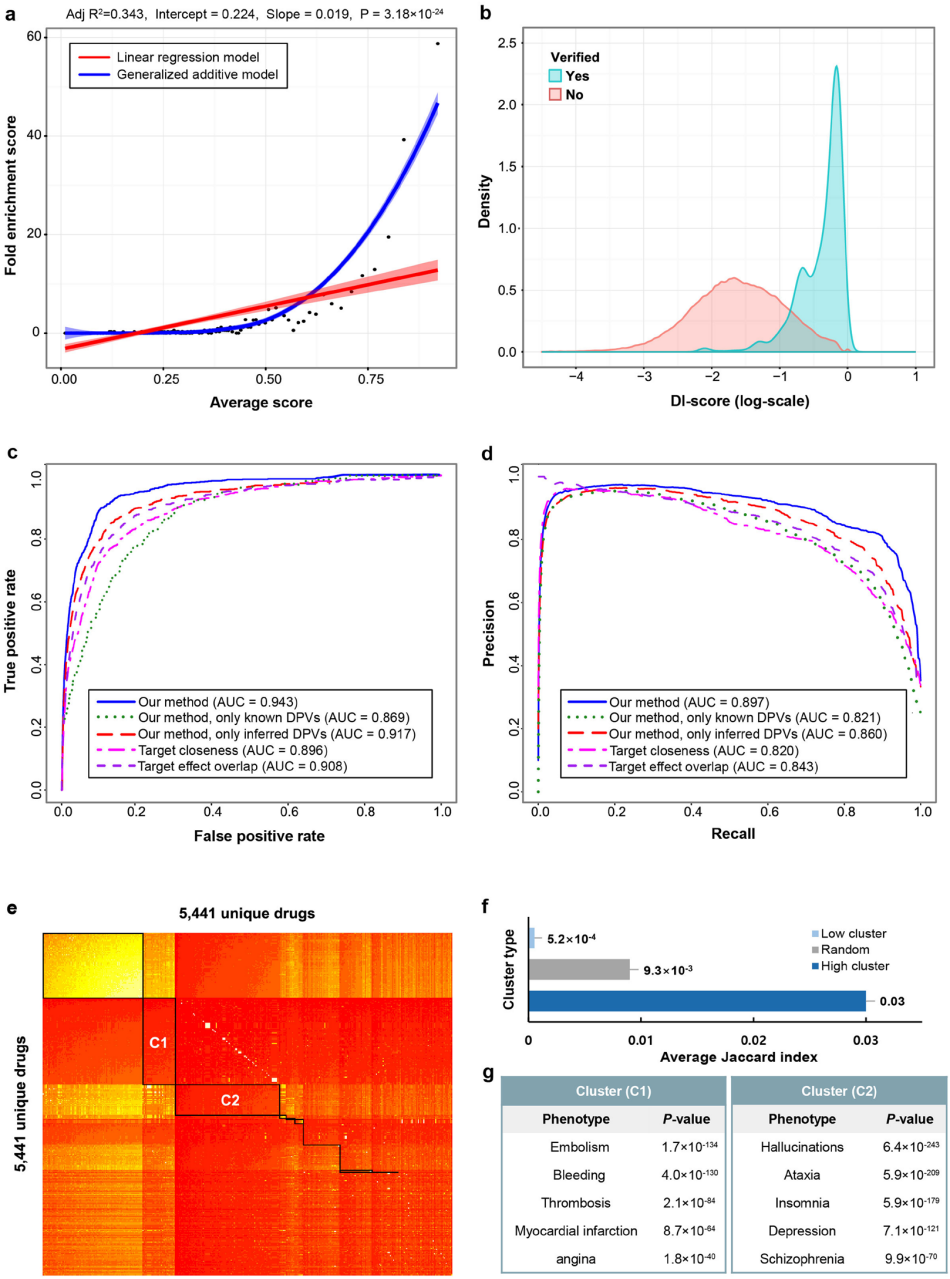
Performance evaluations of the predicted drug interactions. (**a**) All possible drug pairs are ranked and binned into groups by their DI-scores. FE of known pharmacodynamic drug combinations in each bin (y-axis) is plotted against average DI-score (x-axis). Linear regression model (red) and generalized additive model (blue) are used to fit the distribution of FE values (red and blue outlines indicate 95% confidence interval). (**b**) The result of KDE: DI-score distributions show the relative separation between verified (blue) and unverified (red) pharmacodynamic drug combinations. In this plot, the verified pharmacodynamic drug combinations are significantly enriched in higher scores than in lower scores. (**c**) ROC and (**d**) PR curves for our method (blue), our method only with known DPVs (green), our method only with inferred DPVs (red), target closeness method (magenta) and target effect overlap method (purple) to evaluate the performance of DI-score. (**e**) Biclustering of a DI-score matrix. DI-score matrix is generated by calculating DI-score for all possible drug pairs. Red and yellow indicate high and low score values, respectively. DI-score matrix is partitioned into 11 clusters (black box), where the rows and columns are ordered according to the clustering. (**f**) Comparing average Jaccard index between high-scored, low-scored and random clusters. For drug pairs in a cluster, we counted shared phenotypes which are reported in DrugBank and SIDER. Average Jaccard index of each cluster is calculated by averaging Jaccard index of all drug pairs in a cluster. (**g**) Major therapeutic effects (blue) and adverse effects (red) of high scored clusters (Fisher’s test, *p*-value < 0.001).

We performed the FE test to identify the correlation between the DI-score and the likelihood that a drug combination occurs. All possible drug pairs were ranked by the DI-score and binned into groups of 50,000 drug pairs. As a result, as DI-scores increase, the number of drug combinations has markedly increased, especially at top 25% region (Fig. 3a). The result indicates that the drug pairs assigned with high DI-scores are more likely to have drug combinations. We also performed KDE to compare the density distributions of DI-scores between verified and unverified drug combinations. As a result, the distribution of verified drug combination scores was enriched on high score region compared to scores from unverified drug combinations (Fig. 3b). This indicates that verified and unverified drug combinations can be separately detected with the DI-scores. Next, we used AUC scores of ROC curves to examine the quantitative performance of the proposed method in predicting drug combinations. We compared our method with four different cases: (i) using known DPVs only; (ii) using inferred DPVs only; (iii) using target closeness to calculate DDI score by distance between each pair of drug targets on PPI network^21^; and (iv) using target effect overlap to predict DDIs based on similarity between random walk results on PPI network^30^. Importantly, our method, which integrates the information from known and inferred drug-phenotype associations into combined DPVs, exhibited better performance (AUROC = 0.943) than those with single information (AUROC = 0.869~0.917). Also, our method showed better performance than target closeness (AUROC = 0.896) and target effect overlap (AUROC = 0.908) methods (Fig. 3c). However, ROC curves could not reflect the effects of change the proportion of positive to negative instances. In drug combination prediction, large class skew and large changes in class distributions are common, because the negative set of drug combinations is not available. Therefore, many studies have exempted gold standard positive set from all possible drug pairs and used them as the gold standard negative set^11,21,31^. To see the effect due to the class skew, we calculated PR curves. When AUC values from PR curves were compared, our method (AUPR = 0.897 ± 0.004) showed the best performance among the other methods (Fig. 3d). Moreover, we calculated PR curves for different positive/negative ratios to evaluate the performance in the various skewness of dataset and compared it with previous methods (Supplementary Fig. 3). From the result, our method achieved the best performance even with the highly skewed dataset. Finally, we compared our DDI prediction with previous methods, including target closeness, target effects overlap and target connectivity in weighted PPI (Supplementary Fig. 4).

Drug pairs were assigned with high scores when their phenotypic effects were similar. Further analysis was done on whether the sets of drug pairs sharing similar DI-score patterns have similar phenotypes. In order to identify optimal subsets of drug pairs which have high relevance to each other, we performed the biclustering with DI-score matrix to take into account duality between drugs in subspace^32,33^. The result showed that the drug pairs are divided into 11 clusters, including four high-scored and seven low-scored clusters (Fig. 3e). Next, to investigate the difference among high-scored, low-scored and random clusters, we calculated phenotypic similarity for each cluster based on Jaccard index. For a drug pair, a list of associated phenotypes of each drug was gathered from DrugBank and SIDER, and the number of common phenotypes was counted to calculate Jaccard index. From the result, the average Jaccard index of high-scored clusters (JI = 0.03) was markedly higher than the index of low-scored (JI = 5.2 × 10^−4^) and random clusters (JI = 9.3 × 10^−3^) (Fig. 3f). This indicates that high- and low-scored clusters differ strongly with respect to the number of shared phenotypes. Next, we identified major phenotypes of high-scored clusters by performing phenotype enrichment analysis based on Fisher’s exact test. To get Fisher’s test value of each phenotype, the number of drug pairs was counted based on whether they are included in the cluster and whether they have associations with the phenotype. From the result, we found that each cluster has representable phenotypes and that similar phenotypes are enriched to each cluster. For example, phenotypes enriched in cluster 1 (C1 in Fig. 3e) were related to blood clotting disorder or symptoms, including bleeding, thrombosis, pulmonary embolism and myocardial infarction. Whereas phenotypes enriched in cluster 2 (C2 in Fig. 3e) were related to mental and neurological disorders (Fig. 3g). Detailed information of enriched phenotypes of four high-scored clusters can be found in Supplementary Tables 2–5. These results show that sets of drug pairs sharing comparable DI-score patterns have similar phenotypes, providing that our method can be used as a tool to screen sets of drug pairs for specific phenotypes.

Our method predicts drug interactions based on the connectivity and closeness between phenotypes on the phenotypic network, which enables us to find previously undetected drug combinations by target distance-based methods^11,21^. By assigning high scores on drug pairs which have distanced targets but have similar target phenotypes, we can improve prediction coverage of drug combinations (Table 2). For instance, ramipril and irbesartan are being currently prescribed as a drug combination for albuminuria treatment. When measuring the distance between the target genes of these two drugs in the molecular network (average shortest distance = 3.48), they are far away from each other, which is close to random (*p*-value < 0.05). Therefore it is hard to find that these two drugs can be used as a drug combination from target distance-based methods. However, in our method, ramipril and irbesartan receives a high score (DI-score = 0.908) and are proposed as a potential drug combination. Furthermore, we can predict additional phenotypic effects, such as cardiovascular disease, heart condition, hypertension and diabetes nephropathy, which have not been reported in DrugBank and DCDB^34–36^.

**Table 2:** Top 10 drug combinations showing a large difference in rank between our proposed method and target distance-based similarity method.

Agent 1	Agent 2	DI-score	Rank (DI-score)	Rank (Distance-based)
Ramipril	Irbesartan	0.906	47	661
Cyclophosphamide	Amoxapine	0.904	51	620
Telmisartan	Clopidogrel	0.902	57	629
Tiagabine	Gabapentin	0.901	59	689
Lopinavir	Pitavastatin	0.899	65	676
Prednisolone	Mycophenolic acid	0.898	77	543
Sildenafil	Phentolamine	0.892	81	714
Methotrexate	Methylprednisolone	0.889	87	566
Clopidogrel	Cilostazol	0.887	94	811
Sildenafil	(-)-Prostaglandin E1	0.878	127	783

### Analyzing mechanism of action of drug interactions via molecular and phenotypic networks

**Figure 4:**
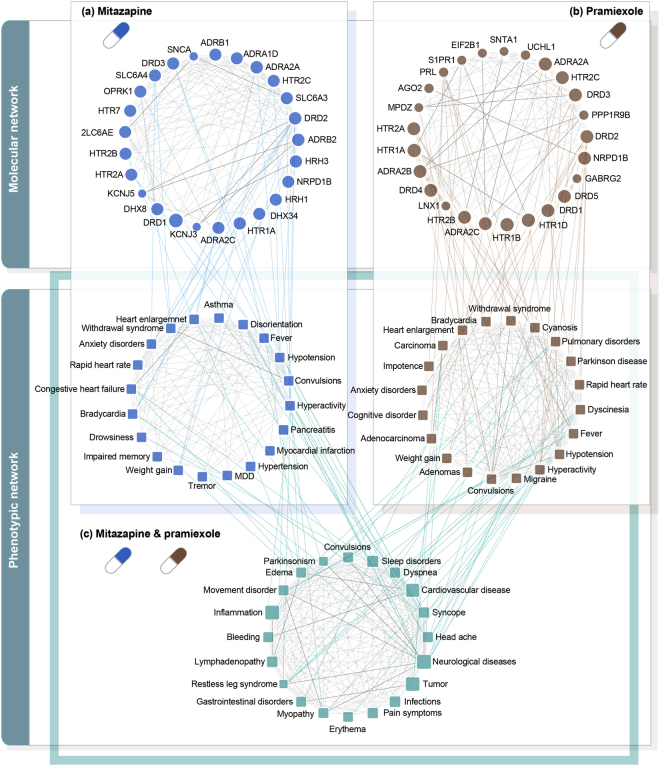
Case study of mirtazapine and pramipexole. (**a**) Propagated effects of mirtazapine (blue) and (**b**) pramipexole (brown) were calculated in molecular and phenotypic networks. Circular composition of the molecular network was constructed by selecting high scored genes (circle) which have interactions between high scored proteins or phenotypes, and the edges were generated by protein-protein interactions. Circular composition of the phenotypic network was constructed by selecting high scored phenotypes (square) which are reported in TWOSIDES, and the edges were generated by phenotype associations of UMLS. The gene nodes were weighted by RWR results, and the phenotype nodes were weighted by the sum of values of associated gene nodes. Edges in the circular network were weighted by shortest path length between nodes. (**c**) Phenotypic effects (light green) of the drug interaction were identified by calculating P-scores based on the candidate phenotypic effects of a drug pair.

Our novel method, which predicts drug effects and interactions from the integrated molecular and phenotypic networks, provides opportunities for better understanding of the molecular mechanisms underlying drug interactions. As an example, an interaction between mirtazapine and pramipexole was predicted with a high score (DI-score = 0.91) of our method (Fig. 4). Mirtazapine is an antidepressant which refines the specificity of effects on the noradrenergic and serotonergic systems by blocking *α*_2_ adrenergic autoreceptors and heteroreceptors. In addition, mirtazapine selectively antagonizes the 5-HT_2_ and 5-HT_3_ serotonin receptors in the central and peripheral nervous system, which are mainly targeted in depression treatment. It also being used as anxiolytic, hypnotic, antiemetic and appetite stimulant^37^. Pramipexole is a non-ergoline dopamine agonist with selective actions at dopamine D_2_ and preferential D_3_ receptor, which are indicated targets for treating Parkinson’s disease and restless legs syndrome^38^. The two drugs, mirtazapine and pramipexole, share 11 common targets. Although their interaction has not been reported in DrugBank and DCDB, recent studies have reported that this drug pair can be used as a drug combination to treat restless legs syndrome and Parkinson’s disease^39,40^. Also, TWOSIDES has reported 99 adverse events including fainting, pain, bleeding and erythema, which cannot be clearly attributed to the individual drugs alone^6^. In our results, therapeutic targets or biomarkers of neurological disorder and mental disorder, such as *DRD1*, *DRD2*, *DRD4*, *HTR2A*, *PRL* and *ADRA2A*, were assigned with high scores in both mirtazapine and pramipexole (Fig. 4a and b). Based on these results, we can consider that neurological disease (P-score = 19.82, rank = 2), parkinsonism (P-score = 9.82, rank = 70) and restless legs syndrome (P-score = 8.19, rank = 140) are related with mirtazapine and pramipexole (Fig. 4c). Furthermore, our method predicted 62 adverse events over 99 reported adverse events, including pain, difficulty breathing, bleeding, erythema and coma with high P-scores (Supplementary Data 3). Next, to understand the MoA of a drug interaction, we investigated whether significantly enriched pathways in both sets of high-scored genes of mirtazapine and pramipexole are associated with our predicted phenotype. For this, genes with the top 10% of propagated drug effects were selected in each drug (Fig. 1a) and pathway enrichment test was performed based on the selected genes by using DAVID tool^41^. Based on these results, we found an intersection of significantly enriched pathways (*p*-value < 0.001) in both drugs. Next, we validated associations between pathways and our predicted phenotypes by PubMed manual curation (Supplementary Data 4). The result shows that a large number of observed pathways are associated with our predicted phenotypes. For example, ‘Neuroactive ligand-receptor interaction’, ‘Dopaminergic synapse’, ‘Serotonergic synapse’, ‘Cholinergic synapse’ and ‘Cocain addiction’ pathways were found to be associated with neurological disease and Parkinson’s disease^42–46^. For inflammation, associated pathways, such as ‘Chemokine signaling pathway’ and ‘cAMP signaling pathway’, were found^47,48^. As another example, an interaction about zoledronic acid and gemcitabine is provided in Supplementary Fig. 5. These case studies show that our method can investigate the MoA of drug interactions based on the propagated effects of drugs and their connectivity on molecular and phenotypic networks.

### Predicted phenotypic effects and their interaction potentials are validated in external literature

To validate the reliability of our method, we confirmed whether the predicted phenotypic effects of drug pairs and their interactions were identified in external literature^49^. We first ranked predicted phenotypic effects of the 894 approved drug combinations by P-scores, and made three independent sets by selecting top 5%, bottom 5% and random 5% phenotypes containing 34,352 drug pair-phenotypic effect associations respectively. For the selected drug pair - phenotypic effects, we counted co-occurrences (*n*_*c*_) from PubMed abstracts, calculated the Jaccard index and conducted the Fisher’s exact test (*n*_*f*_) (Table 3). The average number of co-occurrence of the high-scored set (*n*_*c*_ = 0.87) was 8.7 and 2.8 times larger than the average number of co-occurrence of the low-scored set (*n*_*c*_ = 0.10) and the random set (*n*_*c*_ = 0.31). Also, in order to correct the differences in the frequency of drug pairs and phenotypic effects, the co-occurrence value was normalized by the Jaccard index. From the result, the average value of the high-scored set (JI = 2.9 × 10^−5^) was 3.6 and 2.1 times higher than the values of the low-scored set (JI = 8.1 × 10^−6^) and the random set (JI = 1.4 × 10^−5^). Furthermore, we performed Fisher’s exact test to find the significant associations (*p*-value < 0.001), and the number of significance associations of the high-scored set (*n*_*f*_ = 674) was 7.1 and 3.1 times higher than that of the low-scored set (*n*_*f*_ = 94) and the random set (*n*_*f*_ = 216).

**Table 3:** Literature validation for drug pair-phenotypic effect and drug-drug interaction associations by comparing co-occurrence, Jaccard index and Fisher’s exact test value between high-, low-scored and random sets.

	Drug pair – phenotypic effect			Drug – drug interaction		
	High-scored	Low-scored	Random	High-scored	Low-scored	Random
co-occurrence	0.87	0.10	0.31	1.67	1.9 × 10^−3^	0.24
Jaccard index	2.9 × 10^−5^	8.1 × 10^−6^	1.4 × 10^−5^	1.9 × 10^−4^	3.3 × 10^−7^	5.9 × 10^−6^
Fisher’s exact test^a^	674	94	216	21,380	49	3,109

^a^*p*-value threshold of Fisher’s exact test is 0.001.

Next, we applied above process to predicted drug interactions to validate the DI-score. We ranked all drug pairs by DI-scores, and made three independent sets by selecting top-ranked 5%, bottom-ranked 5% and random 5% drug pairs containing 739,975 drug interactions respectively. For selected drug interactions, the average number of co-occurrence of the high-scored set (*n*_*c*_ = 1.67) was significantly larger than the average number of co-occurrence of the low-scored set (*n*_*c*_ = 1.9 × 10^−3^) and the random set (*n*_*c*_ = 0.24). The average value of the Jaccard index for the high-scored set (JI = 1.9 × 10^−4^) was remarkably higher than the value of the low-scored set (JI = 3.3 × 10^−7^) and the random set (JI = 5.9 × 10^−6^). Finally, in the Fisher’s exact test, the high-scored set (*n*_*f*_ = 21,380) had markedly more significant associations than the low-scored set (*n*_*f*_ = 49) and the random set (*n*_*f*_ = 3,109). As the result shows, we can conclude that both P- and DI-scores can be used as metrics to identify the effects and interactions of drug pairs.

## Discussion

Drug interactions often occur when drugs act on the same or interrelated pathways, resulting in the regulation of biological processes. In this process, unexpected effects may occur due to the complex molecular mechanism of drug interactions. Therefore, identifying phenotypic effects of drug interactions with mechanistic explanation is crucial to increase therapeutic effects while reducing adverse effects. Here, we introduce a phenotype-based approach to predict effects and interactions of drug pairs based on the profiling of systemic effects of drugs. Our analysis of drug effects and their interactions in the molecular and phenotypic networks offers the mechanistic explanation of why phenotypic effects occurred and why drug pair interacts.

In this study, systemic effects of drugs obtained from calculating propagated effects on molecular and phenotypic networks were used as core information in predicting phenotypic effects of drug pairs and their interaction potential. By comparing with random and OFFSIDES results, we confirmed that systemic effects of drugs were successfully predicted with high coverage, and that they were not biased with respect to the number of drug targets and disease associated genes. Based on these results, phenotypic effects of drug pairs were predicted by calculating P-scores considering the connectivity and closeness between phenotypes on the phenotypic network. Most previous methods have focused on predicting potential drug interactions or relations between a certain disease and drug interactions. Even though a few knowledge-based approaches predict adverse effects of drugs and DDIs, they seldom considered the complicated MoA in predicting phenotypic effects of drug interactions. Our predictions attained high coverage of therapeutic effects (63%) and adverse effects (41%) of drugs with the AUROC value of 0.731 and 0.734, respectively. Also, compared with the knowledge-based approach, our method showed better performance in 9 phenotypic effects among 14 phenotypic effects. We further quantified the interplay of drugs by aggregating the P-scores of the drug pair. Compared to the conventional method, our method showed better performance, with high rates of sensitivity and specificity in predicting drug combinations (AUROC = 0.943, AUPR = 0.897). Furthermore, these processes enabled us to find drug interactions previously undetected by target distance-based similarity methods, by allowing high scores on drug pairs which have similar target phenotypes even though their drug targets are distantly located. We also found that drug pairs sharing comparable patterns have similar phenotypes, by applying biclustering on the score matrix of drug interaction. Thus, the drug interaction prediction in this study can be used as a tool to find the sets of drug pairs that are associated with specific phenotypes. Finally, by analyzing candidate phenotypic effects of the previously unknown drug interactions at the molecular and phenotypic networks, possible MoA of those drug interactions could be explained.

However, the given method needs improvements to be directly used as a screening tool in clinical practice. First, the dosage is not taken into an account in the method, although the effects of drug interactions can be varied by different combinations of concentration. Until now, most studies on dose-response relationship have only considered individual drugs, where only a few computational methods have calculated the expected dose-response relationship for drug combinations^2^. Second, limited by the current knowledge of drugs, diseases and interactions of molecule and phenotype, our prediction cannot provide detailed interaction types such as additive, synergistic and antagonistic interactions. Even though our study provides a list of prioritized effects and interactions of drug pairs, the absence of interaction type information is an obstacle to design precise clinical trials. Nevertheless, these limitations can be taken into an account for further experiments or improved computational methods. With these further improvements, our method can be used as a valuable resource in drug development and large-scale clinical trial design, serving as an *in silico* screening tool to provide a list of prioritized drug interactions with phenotypic effects in a cost-effective manner. We believe that the identification of drug interactions with phenotypic effects can be a key factor (i) to provide insights into the underlying molecular and phenotypic mechanisms of the drug interactions and (ii) to extend the combinatorial use of drugs, increasing therapeutic effects while reducing adverse effects.

## Materials and Methods

### Data set

Drug information was collected from DrugBank version 4.3^28^. In this study, we mainly focused on 5,441 drugs including approved and investigational drugs which have at least one target information. Drug targets were collected from DrugBank, DCDB^26^, CTD^50^, MATADOR^51^, STITCH^52^ and TTD^27^ databases, and gene-phenotype associations were collected from CTD database. Drug-phenotype associations were collected from DrugBank, CTD, ClinicalTrials.gov^53^ and DCDB databases by exploiting the MetaMap tool to extract phenotype-related terms^54^. With inputs like narrative text, MetaMap returns a ranked list of Metathesaurus concepts associated with each word of the input text. Among the Metathesaurus concepts categorized in semantic types, we used Metathesaurus concepts assigned to 20 semantic types out of 135 semantic types, which have related phenotypes such as ‘Disease or Syndrome’, ‘Sign or symptom’ and ‘Clinical attribute’ (Supplementary Table 6). Drug side effects were obtained from SIDER^55^ database, and adverse effects of drug pairs were collected from TWOSIDES^6^ database.

A PPI network, including 19,093 nodes and 270,970 edges, was obtained from BioGrid version 3.4.136^56^, and a phenotypic network was taken from Unified Medical Language System (UMLS) in the 2016AA version^25^. UMLS provides integrated information of various terminologies pertaining to biomedicine. The Metathesaurus is the main component of the UMLS which is organized by biomedical concepts, where each distinct concept is assigned to a unique concept identifier (CUI). We collected CUIs with broader (RB), narrower (RN) and other-related (RO) relationships among 11 types of UMLS relationships from MRREL lists, resulting in total 220,104 CUIs and 663,018 relations. For systematic analysis, we integrated molecular and phenotypic networks based on the CODA system which handles various types of biological information^57^.

### Propagation of drug effects from molecules to phenotypes

We constructed a molecular network based on a PPI network and performed RWR algorithm to investigate the propagation of drug effects in the molecular layer. RWR simulates the random walker from its seed nodes and iteratively transmits the node values to the neighbor nodes with the probabilities proportional to the corresponding edge weights^58,59^. First, we assigned initial values to seed nodes in molecule network based on drug-target associations to simulate RWR algorithm. Drug-target associations can be divided into two groups, direct and indirect associations. The biological activity of drugs cause changes in various biological systems by complex interactions with molecular components, and their exact MoA remains largely unknown. Therefore, to expand the coverage of drug effects, we used not only direct (binding) associations, but also indirect associations which can be caused by the changes in the expression of a protein, drug induced phosphorylation or active metabolites of the drug (Supplementary Fig. 1a). The initial values of a direct and indirect association were assigned as 1 and 0.3, respectively. Second, the transition probability from a node to the neighbor node was calculated. We assumed that the transition probability represents the propagated drug effects on the molecular network. The transition probability vector of each node at time step *t* + 1 is defined as equation (1).

1 $${p}_{t+1}=(1-r){W}^{T}{p}_{t}+r{p}_{0}$$

where *r* represents the restarting probability of the random walker at each time step, set to 0.7 in this study. *W* represents the normalized adjacency matrix of the molecular network, *p*_*t*_ is the probability vector of each node at time step *t*, and *p*_0_ represents the initial probability vector. The RWR algorithm simulates the random walker until all nodes reach the steady state (*p*_*t*+1_−*p*_*t*_ < 10^−8^). We then mapped RWR results to phenotypes based on the gene-phenotype associations. In this step, we found all genes which are therapeutic targets or biomarkers of certain phenotypes and mapped the sum of these gene values, which were obtained from RWR results, to the corresponding phenotypes. Through this process, we obtained a list of phenotype values for each drug.

### Filter effective phenotypes from a large number of candidates

Although a phenotype value calculated from propagated drug effects is high, it may not mean that the drug is highly related to the phenotype. When there are many phenotype associated genes, or when a drug has a large number of target proteins, overall phenotype values get increased stochastically. To overcome this problem, we generated random DPVs and compared them with a list of inferred phenotype values to select phenotypes with significant values. A random DPV was generated by randomly selecting drug’s targets while fixing the number of target proteins. For each drug, 1,000 random DPVs were generated, and phenotypes with an empirical *p*-value lower than 0.01 were selected. The empirical *p*-value was calculated as equation (2).

2 $$p=(r+1)/(n+1)$$

where *n* is the number of random DPVs and *r* is the number of DPV values that are larger than the phenotype value, respectively^60^. Value one was assigned on phenotypes with the *p*-value lower than 0.01, and zero on the others. From this process, inferred DPVs were generated with filtered effective phenotypes from a large number of inferred candidates of drug effects. Although phenotypic effects of drugs were extracted from the molecular network, there may have been an omission of some drug-phenotype associations due to the incomplete information such as pathophysiology information about diseases, protein interaction and molecular mechanisms of a drug. Therefore, the combined DPVs were generated by combining both known and inferred DPVs which cover the large amount of known and unexpected drug-phenotype associations (Fig. 1b).

### Predicting phenotype-specific interaction of drug pairs

A set of candidate phenotypic effects of a drug pair was defined as a union of phenotypes in combined DPVs. There are hundreds of phenotypes in each combined DPV, so we prioritized phenotypes by P-scores to filter out meaningless phenotypes. For this, we mapped phenotypes of the combined DPVs to the phenotypic network. The P-score of a phenotype is calculated by considering phenotype pairs containing this specific phenotype among the whole pairs with the closeness in the phenotypic network as equation (3)^61,62^.

3 $${\rm{P}}-{{\rm{score}}}_{{d}_{1}{d}_{2}}(c)=\sum _{p\in P}{e}^{-d(c,p)}({v}_{{1}_{c}}{v}_{{2}_{p}}+{v}_{{1}_{p}}{v}_{{2}_{c}})$$

where *c* is the phenotype of interest, and *P* is the set of phenotypes in the combined DPV. *d*(*c*,*p*) is the shortest path length between phenotype nodes c and p in the phenotypic network. Therefore, *e*^−*d*(*c*, *p*)^ is increased when two phenotypes are closely located in the phenotypic network. ${v}_{{i}_{j}}$ is the value of phenotype *j* in the combined DPV of drug *i*. If the phenotype belongs to both combined DPVs, and the phenotype is closely located to the phenotype set of the opposite combined DPV in the phenotypic network, then the phenotype is given a high value. Conversely, if the phenotype belongs to less than one combined DPV, and there is no connection between the phenotype and the phenotype set of the other combined DPV, then the phenotype is given a minimum value (zero).

### Phenotype-based prediction of drug interactions

The interaction potential between each drug pair was quantified based on phenotypic effects of the drug pair. The underlying hypothesis of this study was that a drug pair can interact with each other if they have similar phenotypic effects. Therefore, we defined an interaction potential of a drug pair as a sum of P-scores of phenotype candidates as equation (4).

4 $${\rm{IP}}({d}_{1},{d}_{2})=\sum _{p\in P}{\rm{P}}-{{\rm{score}}}_{{d}_{1},{d}_{2}}(p)$$

If a drug pair has lots of similar phenotypic effects, the number of phenotypes with a high P-score will increase and the interaction potential will be given a high value. Conversely, if there are no shared phenotypes between a drug pair, the interaction potential will be given a low value. Next, the interaction potential was normalized by dividing it by the geometric mean of the interaction potential values, which were obtained by calculating each interaction potential against itself. Finally, the DI-score is calculated as equation (5)

5 $${\rm{DI}}-{\rm{score}}({d}_{1},{d}_{2})=\,\frac{IP({d}_{1},{d}_{2})}{\sqrt{IP({d}_{1},{d}_{1})\cdot IP({d}_{2},{d}_{2})}}$$

The value of the DI-score is zero (min) when combined DPVs have no shared phenotypes and phenotypes between combined DPVs are disconnected in the phenotypic network, and the value is one (max) when combined DPVs have the same phenotypes. DI-score has following characteristics: (i) the DI-score is related to the closeness between phenotype components; (ii) the DI-score is independent of the size of combined DPVs; and (iii) the maximum DI-score is assigned when combined DPVs are identical, no matter how many phenotypes they share (Supplementary Fig. 6).

### Performance evaluation

To measure the performance of our method in predicting drug combinations and DDIs, we collected 1,093 known drug combinations from DCDB^26^ and TTD^27^ databases and 29,074 known DDIs from DrugBank^28^ and KEGG^29^, which were used as the gold standard positive set. Since the negative set of drug combinations and DDIs is not available, we exempted gold standard positive set from all possible drug pairs and used them as the gold standard negative set. These sets were used to calculate the ROC, FE and KDE. Moreover, unlike the ROC curve, the PR curve is very sensitive to the imbalance of positive/negative ratio^63^. Therefore, we generated negative sets by considering the various negative/positive ratios in samples (1:10, 1:100, 1:500, 1:1000), highlighting the difference in performance measures which might be lost in the ROC curve analysis (Fig. 2d). To obtain robust AUC score estimates, we performed our method 10 times by randomly selecting different negative sets. We then averaged the resulting AUC scores, and benchmarked the AUC performance against the performance of previous methods. Next, we performed FE test to evaluate whether the drug pairs identified by the high similarity score are more likely to result in drug combinations. In the FE test, all possible drug pairs were ranked by the DI-score and binned into groups of 50,000 drug pairs. The FE is defined as equation (6).

6 $$Fold\,enrichment=\frac{m/n}{M/N}$$

where *m* is the number of known drug combinations in each bin among all known drug combinations *M*, and *n* is the number of drug pairs in each bin among the total possible drug pairs *N*. Linear regression model and generalized additive model were used to fit the distribution of FE values^64^. In addition, we estimated probability density function of positive and negative set scores by KDE to compare the distribution of positive and negative sets^65^.

In order to identify drug pairs which have a high relevance to each other, we performed the biclustering with DI-score matrix based on Plaid model in R^66^. Biclustering is used to find a subset of columns and rows with a high similarity scores in a table, which is composed of different rows and columns. However, as in this paper, biclustering can be applied to symmetric matrices where column and row are the same. If a one-sided clustering algorithm is applied in our result, drugs will be clustered considering the similarity of interaction scores across all 6,499 drugs. However, groups of patterns found in drug pair matrix are not homogeneous across all the drug pairs. Rather, only a subset of the drug pairs possesses these groupings. Therefore, biclustering was performed to find an optimal co-cluster with a high interaction score between drugs in the subset of drugs.

The phenotype enrichment analysis of each cluster is performed by Fisher’s exact test, and two-sided p-value and odds ratio are used to evaluate the strength of the enrichment of phenotypes among the clustered and un-clustered drug pairs.

## Electronic supplementary material

Supplementary information

Dataset 1

Dataset 2

Dataset 3

Dataset 4
