# Supplementary material for: In silico profiling of systemic effects of drugs to predict unexpected interactions
Source: Sci Rep. 2018 Jan 25;8:1612. doi: 10.1038/s41598-018-19614-5 (PMC5785495; doi:10.1038/s41598-018-19614-5)
Supplement: Supplementary file 1 — Supplementary information [file 41598_2018_19614_MOESM1_ESM.pdf]

Supplementary information for

In silico profiling of systemic effects of drugs to predict unexpected interactions

Sunyong Yoo[1], Kyungrin Noh[2], Moonshik Shin[1], Junseok Park[1], Kwang-Hyung Lee[1*], Hojung Nam[3*] and Doheon Lee[1,2*]

[1]Department of Bio and Brain Engineering, Korea Advanced Institute of Science and Technology (KAIST), Daejeon 34141, Republic of Korea

[2]Bio-Synergy Research Center, Daejeon 34141, Republic of Korea

[3]School of Electrical Engineering and Computer Science, Gwangju Institute of Science and Technology (GIST), Gwangju 61005, Republic of Korea

*Corresponding author

Inventory

Supplementary Figures

Supplementary Tables

Supplementary References

Supplementary Figures

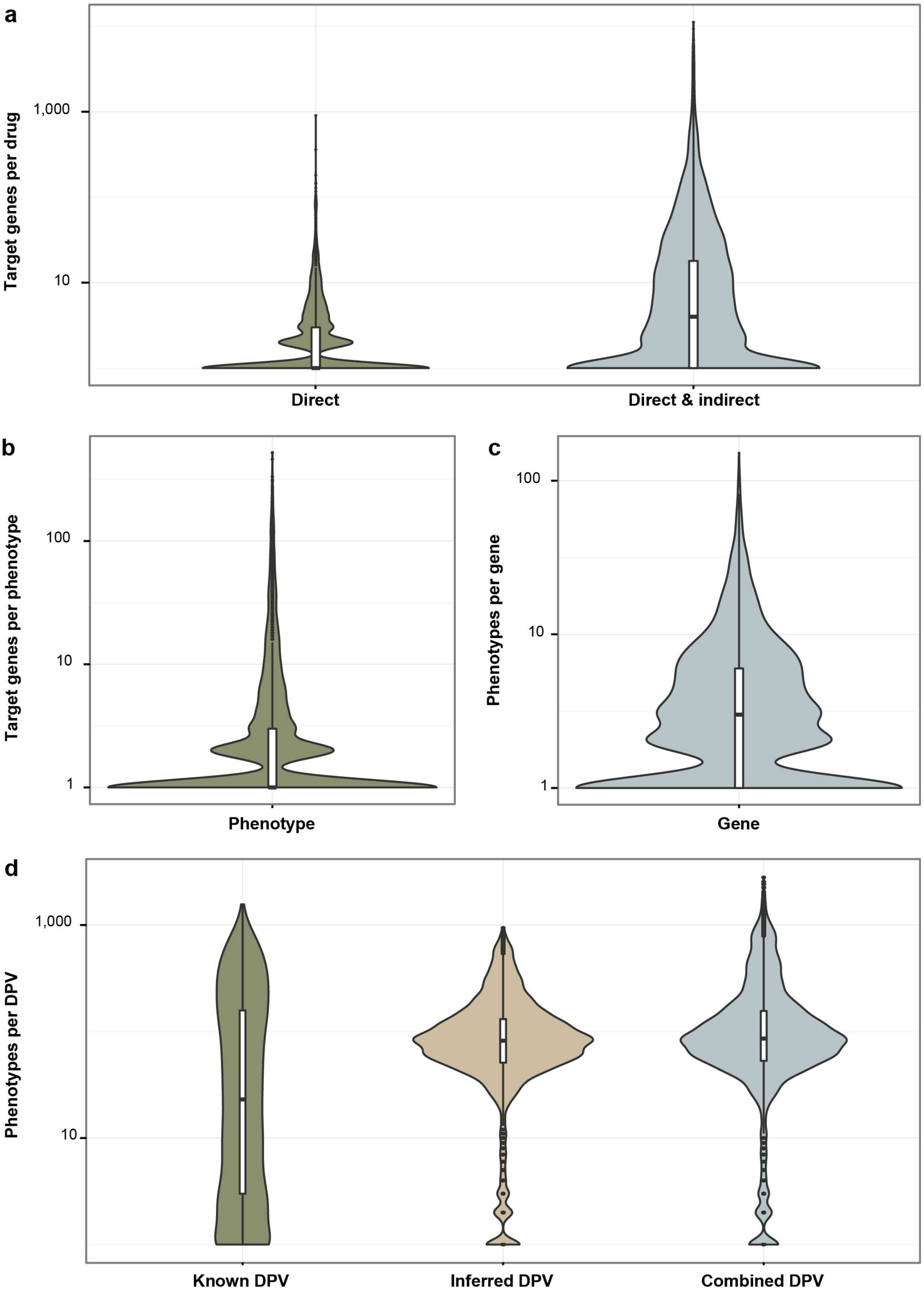

Supplementary Figure 1. The distribution of the number of targets associated with drugs, genes associated with phenotypes and phenotypes associated with drugs. (a) The distribution of direct and indirect target genes per drug. The mean of direct target genes is 3.7 and indirect target genes is 51.7. **(b)** The distribution of target genes per phenotype. The mean of target genes per phenotype is 5.1. **(c)** The distribution of phenotypes per gene. The mean of phenotypes per gene is 11.4. **(d)** The distribution of phenotypes per known, inferred and combined DPVs. The mean of phenotypes of known, inferred and combined DPVs is 57.4, 116.1 and 160.8, respectively.

Prediction of therapeutic and adverse effects of drug interactions

Our method predicted at least one known phenotypes in 829 drug combinations (86%). Additionally, candidate phenotypic effects covered 1,608 phenotypes (63%) among 2,535 phenotypes. However, per drug combination, there exists 746 phenotype candidates in average, and for all 829 drug combinations, there are 618,944 phenotype candidates. Similarly, for adverse effects prediction, 161,069 phenotypes (41%) were predicted from 14,714 drug interactions (84%) using adverse effects information of TWOSIDES as a silver standard. Evaluation of adverse effect prediction is generally not straightforward, because there are no gold standard of verified adverse effects of drug interactions covering large number of combinations and diseases. Therefore, we used TWOSIDES as a silver standard, which mined adverse effects of drug interactions from the ADR reports.

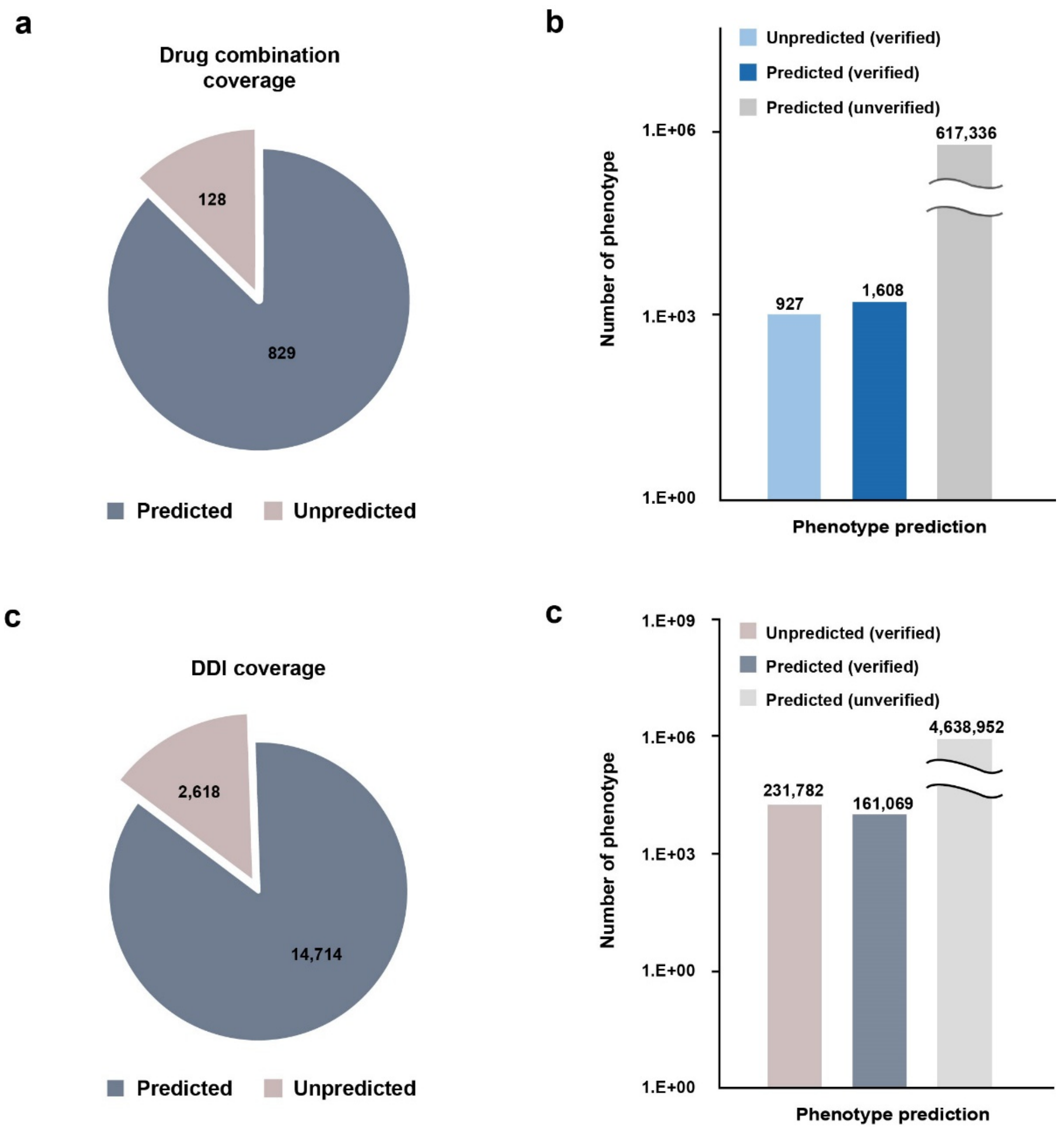

Supplementary Figure 2. Coverage of drug combinations and DDIs in predicting their phenotypes. (a) Coverage of drug combinations in predicting their phenotypes. **(b)** Comparison of the number of phenotypes between predicted and unpredicted cases. Unpredicted (verified) bar represents the number of verified phenotypes which were unpredicted by our method. Predicted (verified) bar represents the number of verified phenotypes which were predicted by our method. Predicted (unverified) bar represents the number of unverified phenotypes which were predicted by our method. **(c)** Coverage of DDIs in predicting their phenotypes. **(d)** Comparison of the number of phenotypes between predicted and unpredicted cases. Unpredicted (verified) bar represents the number of verified phenotypes which were unpredicted by our method. Predicted (verified) bar represents the number of verified phenotypes which were predicted by our method. Predicted (unverified) bar represents the number of unverified phenotypes which were predicted by our method.

Comparison with previous methods

We compared our prediction performance with previous methods (Supplemental Fig. 3 and 4).

Target closeness

The similarity for a query drug pair was calculated based on the distances between each pair of drug targets. Distances were calculated using the shortest paths algorithm on the PPI network. Shortest path between any two targets was first searched using Dijkstra's algorithm. The similarity was calculated by considering distances of all target pairs (Gottlieb et al, 2012; Li et al, 2015). We evaluated the performance in predicting DDIs. We used 911 pharmacodynamic drug combinations as a gold-standard dataset for comparison with our method.

Target effect overlap

The similarity for a query drug pair was calculated by considering propagated drug effects on the PPI network. To simulate the signal propagation, random walk with restart algorithm was applied. Based on the random walk results of query drugs, protein scores were calculated which represents the overlapping influence on the same proteins between drugs. Finally, DDI score was calculated by the summation of the protein score of all proteins which represents the interference score between drugs as determined. For a fair comparison with the Park et al. study, we used 13,357 pharmacodynamic DDIs as a gold-standard which was used in Park et al. study (Park et al, 2015).

Target connectivity in weighted PPI (Huang's study)

Instead of using raw information of PPI network, weighted PPI network was used which is constructed by Pearson's correlation coefficient of encoding genes' expression profile across 79 human tissues (Huang et al, 2013; Su et al, 2004). Then a target-centered system was constructed for each drug, which includes drug targets and their first-step neighboring proteins in the PPI network. Finally, system connection score (S-score) was calculated to describe the connection between two target-centered systems in the PPI network. For a fair comparison with the Huang et al. study (Huang et al, 2013), we used 11,406 pharmacodynamic DDIs as gold-standard positive set, and 217,743 pharmacodynamic DDI candidates as a gold-standard negative set, which was used in Huang et al. study.

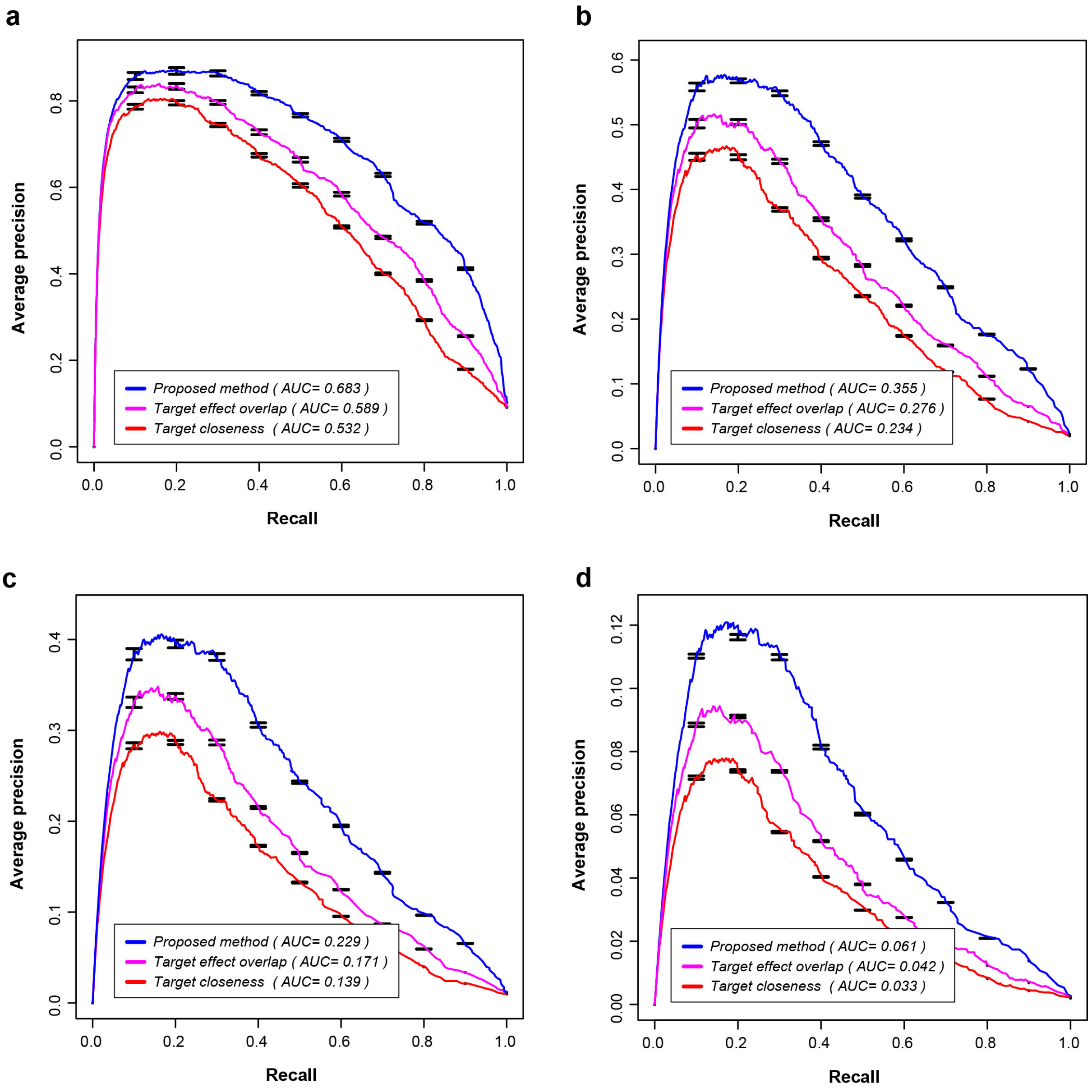

Supplementary Figure 3. PR curves to evaluate the prediction performance of DI-score in the various skewness of dataset. We consider five different positive/negative ratios, including **(a)** 1:10, **(b)** 1:100, **(c)** 1:500 and **(d)** 1:1000, to evaluate that our model has good performance comparing with previous methods even in the large skewedness data set.

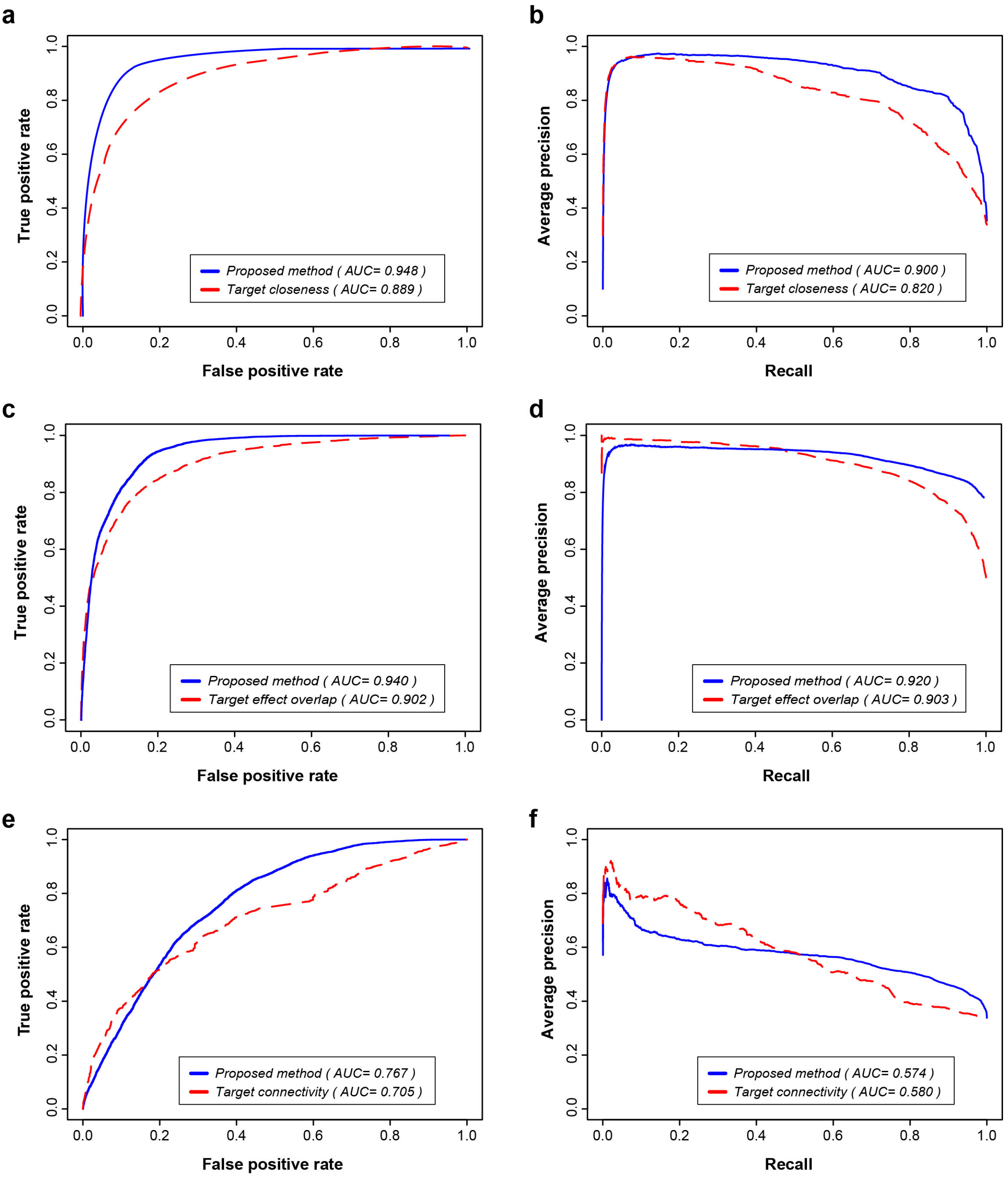

Supplementary Figure 4. Performance comparison with previous methods in DDI prediction. (a) ROC and **(b)** PR curves for our method (blue) and target closeness method (red). **(c)** ROC and **(d)** PR curves for our method (blue) and Target connectivity in weighted PPI method (red). **(e)** ROC and **(f)** PR curves for our method (blue) and target effect overlap method (red).

Case study

Zoledronic acid or zoledronate is a bisphosphonic acid which is an inhibitor of osteoclastic bone resorption (Aronhime & Lifshitz-Liron, 2009). Zoledronic acid is used to prevent osteoporosis and skeletal fractures, particularly in patients with cancers such as multiple myeloma and prostate cancer. It can also be used to treat hypercalcemia, particularly hypercalcemia of malignancy, and pain from bone metastases. Gemcitabine, a nucleoside analog of deoxycytidine, is an antineoplastic anti-metabolite which mainly inhibits DNA synthesis through interfering with DNA chain elongation and indirectly inhibits DNA replication through depleting nucleoside salvage pathway, resulting in gemcitabine-induced cell death. Gemcitabine is clinically used to treat a variety of solid tumors, particularly pancreatic, bladder and non-small cell lung cancers. These two drugs have 52 common targets, and their interaction was assigned with a high score from our method (DI-score=0.9). Although their interaction has not been reported in DrugBank and DCDB, recent studies have reported that this drug pair can be used as a drug combination to treat pancreatic cancer and gastric cancer, and to enhance antitumor activity (Cox et al, 2006; Koto et al, 2010; Trojan et al, 2005; Zhao et al, 2012). Also, TWOSIDES reported 84 adverse events including bronchitis, hydronephrosis, anorexia and arthropathy, which cannot be clearly attributed to the individual drugs alone (Tatonetti et al, 2012). In our results, therapeutic targets or biomarkers of tumor and carcinoma, such as TP53, MCM2, EGFR, IFNG and CDKN1A, were assigned with high scores in both zoledronic acid and gemcitabine. Based on these results, we predicted that tumor (P-score=15.19, rank=1), carcinoma (P-score=9.19, rank=13) and pancreatic disorder (P-score=7.43, rank=74) are related with zoledronic acid and gemcitabine. Furthermore, our method predicted 28 adverse events over 84 reported adverse events, including edema, dyspnea, cardiomyopathy, mucositis and anorexia with high P-scores.

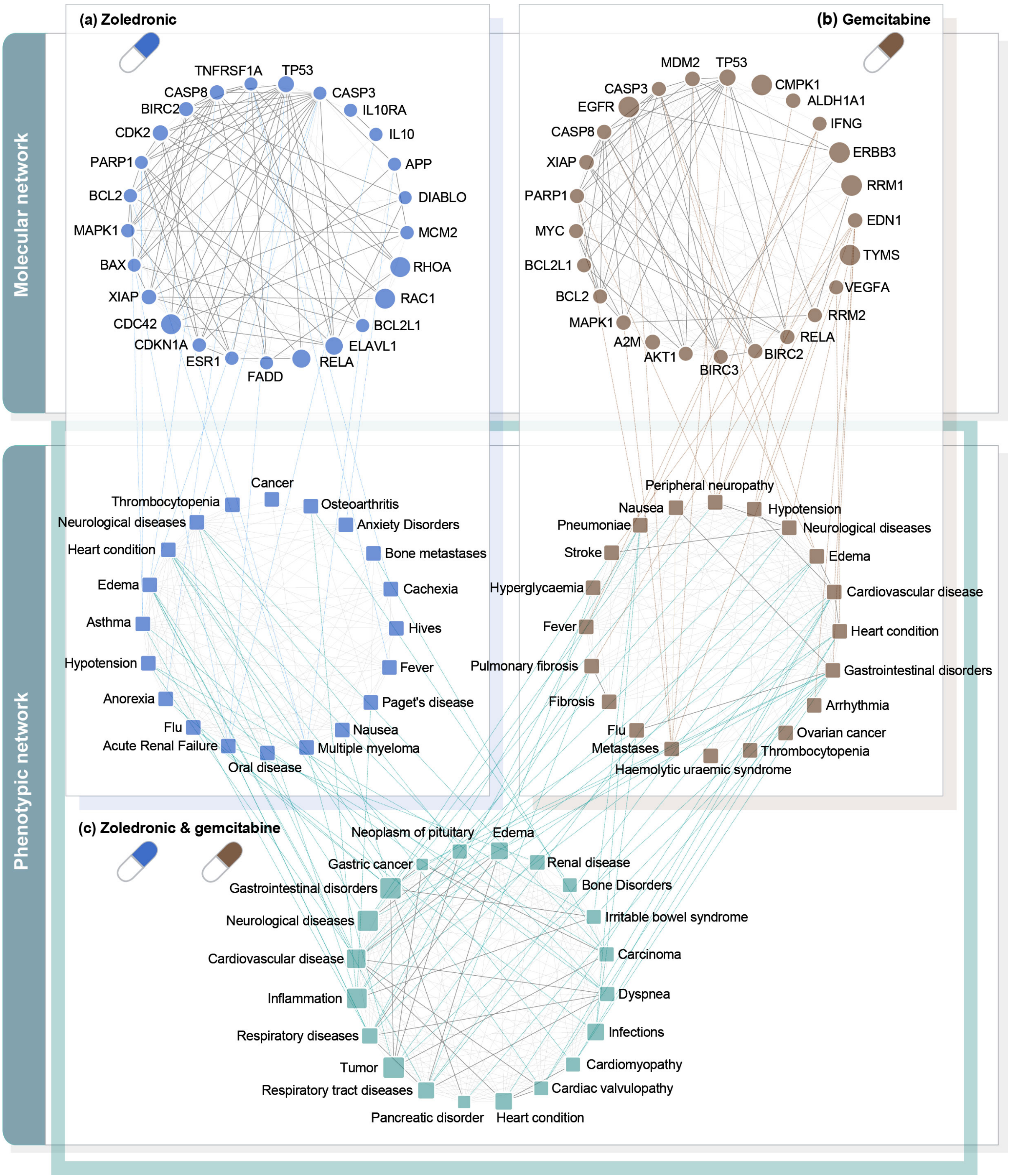

Supplementary Figure 5. Case study of zoledronic and gemcitabine. (a) Propagated effects of zoledronic (blue) and **(b)** gemcitabine (brown) were calculated in molecule and phenotype space. Circular network of molecule space was constructed by selecting high scored genes (circle) which have interactions between high scored proteins or phenotypes, and their edges were generated by protein-protein interactions. Circular network of phenotype space was constructed by selecting high scored phenotypes (square) which reported in TWOSIDES, and their edges were generated by phenotype associations of UMLS. The gene nodes were weighted by RWR results, and the phenotype nodes were weighted by the sum of values of associated gene nodes. Edges in the

circular network were weighted by shortest path length between nodes. **(c)** Hidden effects (light green) of drug interaction were identified by calculating connectivity score based on candidate phenotypic effects of a drug pair.

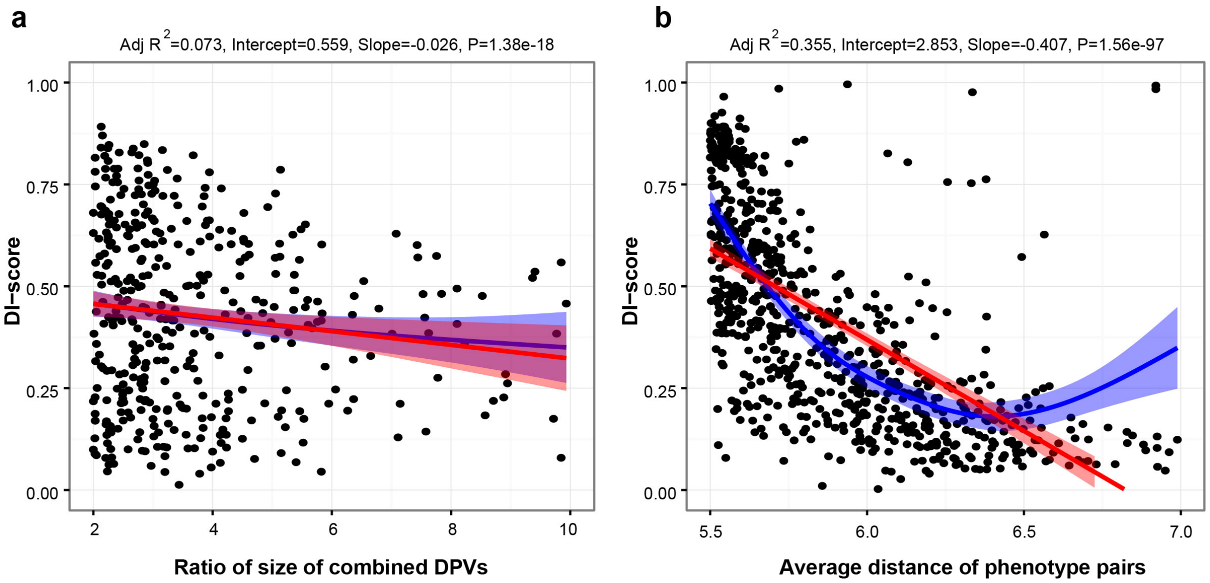

Supplementary Figure 6. Assessment of the DI score. (a) DI-score of all possible drug pairs (y-axis) plotted against ratio of size of combined DPVs (x-axis). The DI-score is robust even when the combined DPV size is significantly different. **(b)** DI-score of all possible drug pairs (y-axis) plotted against average distance of phenotype pairs (x-axis). Red line is linear regression model and blue line is generalized additive model. High DI-score was assigned to a drug pair which has the low average distance of phenotype pairs.

Supplemental Tables

Supplementary Table 1. The relationship between precision, recall and F1 score according to *p*-value threshold.

p-value	Therapeutic effects			Side effects			Potential candidates		
	Precision	Recall	F1	Precision	Recall	F1	Precision	Recall	F1
0.1	0.011	0.569	0.021	0.022	0.515	0.042	0.192	0.854	0.312
0.01	0.033	0.339	0.060	0.079	0.256	0.120	0.381	0.557	0.452
0.001	0.042	0.101	0.059	0.097	0.127	0.109	0.481	0.332	0.393

Supplementary Table 2. Summary of phenotype enrichment analysis for cluster 1. The result shows that a large number of phenotypes enriched in cluster 1 were related to blood clotting disorder or symptoms, including bleeding, thrombosis, pulmonary embolism and myocardial infarction.

**Cluster 1**			
**UMLS ID**	**Name**	***p*-value**	**Odds ratio**
C1704212	Embolism	1.74×10^{-134}	520.3887
C0019080	Bleeding	4.03×10^{-130}	47.41597
C0034065	Pulmonary Embolism	1.14×10^{-95}	253.7189
C0040053	Thrombosis	2.13×10^{-84}	137.0602
C2926063	Myocardial infarction	8.71×10^{-64}	38.38053
C0002965	Angina, Unstable	1.84×10^{-40}	233.5742
C0948089	Syndromes, Acute Coronary	5.89×10^{-38}	167.7413
C0042487	Thrombosis of vein NOS	1.77×10^{-33}	187.3592
C0004238	Atrial Fibrillation	2.90×10^{-29}	38.36223
C0038454	Stroke	4.68×10^{-20}	46.65419
C0027497	Nausea	3.36×10^{-14}	0.392677
C0040038	Thromboembolic disorders	6.41×10^{-13}	288.5671
C0018681	Head ache	3.97×10^{-10}	0.096579
C0149871	Deep vein thrombosis	2.35×10^{-9}	381.2776
C1328851	blood forming organ	2.35×10^{-9}	381.2776
C0036572	Convulsions	6.10×10^{-9}	0.15335
C0011991	Diarrhea	1.60×10^{-8}	0.181123
C0009938	Bruising	3.35×10^{-8}	159.4687
C0007780	Cerebral embolism	7.51×10^{-7}	238.9047
C1861172	Venous Thromboembolism	1.05×10^{-6}	59.92505
C0155626	Acute myocardial infarction	3.69×10^{-6}	42.59868
C0042963	Vomiting	3.96×10^{-6}	0.512615
C0040034	Thrombocytopenia	1.04×10^{-5}	8.055606
C0020649	Hypotension	1.37×10^{-5}	0.340431
C2979982	Vaginal bleeding	2.59×10^{-5}	480.3123
C3537184	NSTEMI	2.59×10^{-5}	480.3123
C0003842	Arterial	2.59×10^{-5}	480.3123
C0002994	Angioedema	6.28×10^{-5}	19.96894
C2678378	Hypotension, severe	1.31×10^{-4}	16.38577
C3538872	STEMI	1.55×10^{-4}	136.9544

Supplementary Table 3. Summary of phenotype enrichment analysis for cluster 2. The result shows that a large number of phenotypes enriched in cluster 2 were related to mental and neurological disorders.

**Cluster 2**			
**UMLS ID**	**Name**	***p*-value**	**Odds ratio**
C0018524	Hallucinations	6.45×10^{-243}	8.260395
C0004134	Ataxia	5.95×10^{-209}	30.98056
C0002962	Angina pectoris	2.20×10^{-206}	17.64798
C0042029	Urinary tract infections	2.92×10^{-204}	12.29938
C0917801	Insomnia	5.98×10^{-179}	4.619285
C0006142	Cancer of the Breast	1.96×10^{-163}	10.75196
C0003123	Anorexia	1.23×10^{-147}	19.83453
C0853697	Neutropenia	3.69×10^{-132}	22.23174
C0011860	Diabetes mellitus, type 2	9.09×10^{-129}	4.098926
C0344315	Depression	7.19×10^{-121}	3.371811
C0235063	Respiratory depression	1.07×10^{-114}	6.806543
C0086439	Decreased activity	1.60×10^{-102}	38.48687
C0013404	Dyspnea	4.28×10^{-96}	6.05586
C0004096	Asthma	4.47×10^{-95}	5.076021
C0022658	Renal disease	5.50×10^{-95}	17.36176
C0003811	Arrhythmia	7.61×10^{-92}	6.114674
C0003467	Anxiety	2.80×10^{-91}	7.261351
C0011175	Dehydration	3.78×10^{-89}	88.21761
C1963252	Tremor	4.87×10^{-85}	11.4896
C0018801	Cardiac failure	6.12×10^{-81}	19.05492
C0021368	Inflammation	1.81×10^{-78}	21.50287
C1961131	Cough	3.54×10^{-73}	8.171448
C0020621	Hypokalaemia	9.79×10^{-73}	53.11573
C2700617	Irritability	2.91×10^{-72}	6.82745
C0036341	Schizophrenia	9.90×10^{-70}	3.761619
C0014544	Epilepsy	1.62×10^{-67}	6.86689
C0857121	hypertensive	5.11×10^{-66}	144.5169
C2678378	Hypotension, severe	1.60×10^{-63}	30.33367
C0006266	Bronchospasm	3.87×10^{-63}	10.61081
C0002871	Anaemia	1.00×10^{-61}	6.817989

Supplementary Table 4. Summary of phenotype enrichment analysis for cluster 3. The result shows that a large number of phenotypes enriched in cluster 3 were related to adverse reactions of psychotropic drugs, including convulsions, hypotension, coma and dizziness.

**Cluster 3**			
**UMLS ID**	**Name**	***p*-value**	**Odds ratio**
C0036572	Convulsions	1.31×10^{-174}	18.52519
C0020649	Hypotension	1.69×10^{-118}	15.53507
C0009421	Coma	1.21×10^{-106}	38.21924
C0012833	Dizziness	2.06×10^{-68}	17.96044
C0042963	Vomiting	1.13×10^{-50}	14.44693
C0027497	Nausea	9.57×10^{-50}	11.78933
C0043352	Dry mouth	1.58×10^{-49}	34.88631
C0003811	Arrhythmia	7.58×10^{-49}	46.87661
C0039231	Rapid heart rate	5.30×10^{-40}	21.92858
C0013604	Edema	5.88×10^{-39}	25.4126
C0015967	Fever	1.15×10^{-36}	10.07809
C0020538	Hypertension	1.38×10^{-35}	7.318543
C0015672	Fatigue	8.33×10^{-29}	9.020123
C0026961	Mydriasis	1.25×10^{-27}	48.07321
C0013404	Dyspnea	3.40×10^{-26}	19.29582
C1963252	Tremor	3.40×10^{-26}	39.87442
C0237314	Irregular heartbeat	2.33×10^{-24}	49.11885
C0149931	Migraine	1.40×10^{-21}	26.38367
C0006266	Bronchospasm	3.77×10^{-20}	36.66379
C0424295	Hyperactivity	4.73×10^{-20}	80.21936
C0242422	Parkinsonism	8.68×10^{-20}	183.2454
C0026837	Rigidity, Muscular	1.58×10^{-17}	183.2454
C2830004	Somnolence	1.83×10^{-17}	128.3286
C0009443	Common Cold	3.28×10^{-17}	34.65524
C0428977	Bradycardia	1.77×10^{-15}	6.978694
C0917801	Insomnia	1.98×10^{-15}	6.147839
C0036974	Shock	5.36×10^{-15}	14.4737
C0018790	Cardiac arrest	5.36×10^{-15}	22.65064
C0264886	Cardiac dysrhythmias	7.29×10^{-15}	117.414
C0041657	Loss of consciousness	7.62×10^{-15}	35.22932

Supplementary Table 5. Summary of phenotype enrichment analysis for cluster 4. The result shows that a large number of phenotypes enriched in cluster 4 were related to cancer related terms, including renal cell carcinoma, chronic myelogenous leukemia, gastric carcinoma and Ph$^+$ acute lymphoblastic leukemia.

**Cluster 4**			
**UMLS ID**	**Name**	***p*-value**	**Odds ratio**
C0011991	Diarrhea	1.73×10^{-26}	7.016038
C0027497	Nausea	4.28×10^{-26}	3.622098
C0015230	Skin rash	2.35×10^{-25}	31.3007
C0040034	Thrombocytopenia	7.56×10^{-21}	63.37191
C0280100	Solid tumors	3.33×10^{-20}	19.59643
C0042963	Vomiting	7.34×10^{-15}	3.398458
C0003862	Arthralgia	3.08×10^{-14}	105.8498
C0007134	Renal cell carcinoma	1.05×10^{-12}	108.8218
C0005699	Blast phase chronic myelocytic Leukemia	8.82×10^{-11}	727.052
C0002170	Loss of hair	1.13×10^{-10}	92.43232
C0009806	Constipation	6.28×10^{-10}	17.52675
C0013604	Edema	2.67×10^{-7}	13.21847
C0024902	Mastalgia	1.32×10^{-6}	2047.038
C0043096	Loss of weight	1.24×10^{-5}	29.8743
C0015967	Fever	1.50×10^{-5}	5.737285
C0027947	Neutropenia	1.95×10^{-5}	26.61656
C0033860	Psoriasis	2.04×10^{-5}	15.85787
C0232462	Decrease in appetite	2.64×10^{-5}	303.6816
C0023473	Chronic myelogenous leukemia	2.64×10^{-5}	303.6816
C0001144	Acne vulgaris	2.86×10^{-5}	54.12944
C0151908	Dry skin	7.72×10^{-5}	38.44163
C0242383	Age related macular degeneration	1.38×10^{-4}	125.6007
C0041834	Erythema	1.06×10^{-3}	43.43872
C0006118	Tumor of the brain	1.88×10^{-3}	726.789
C1963120	Gynecomastia	1.88×10^{-3}	726.789
C0006145	Breast disease	1.88×10^{-3}	726.789
C0699791	Gastric carcinoma	1.88×10^{-3}	726.789
C0016034	Fibrocystic breast disease	1.88×10^{-3}	726.789
C1960397	Ph$^+$ acute lymphoblastic leukemia	4.22×10^{-3}	264.9805
C0020538	Hypertension	5.47×10^{-1}	1.364797

Supplementary Table 6. Phenotype-related UMLS semantic types. The UMLS concepts are categorized into 133 semantic types which represent the category of a concept. Among them, following 20 semantic types are selected to be related with phenotypes, and used as filtering criteria for phenotype terms. For example, plain text '*Treatment of sore throat, colitis, dysentery*' is mapped into four concepts, *Treatment* (C0039798; ftcn), *Sore throat* (C0031350; dsyn), *Colitis* (C0009319; dsyn) and *Dysentery* (C0013369; dsyn), in which semantic type 'ftcn' means functional concept and 'dsyn' means disease or syndrome. Because the filtering criteria include 'dsyn' but not 'ftcn', the resulting phenotype terms are *Sore throat* (C0031350), *Colitis* (C0009319) and *Dysentery* (C0013369). Consequentially, the phenotype information in plain text was extracted.

Abbreviation	Semantic type
acab	Acquired Abnormality
anab	Anatomical Abnormality
biof	Biologic Function
cgab	Congenital Abnormality
comd	Cell or Molecular Dysfunction
dsyn	Disease or Syndrome
emod	Experimental Model of Disease
fndg	Finding
inpo	Injury or Poisoning
lbtr	Laboratory or Test Result
menp	Mental Process
mobd	Mental or Behavioral Dysfunction
neop	Neoplastic Process
patf	Pathologic Function
phsf	Physiologic Function
sosy	Sign or Symptom
clna	Clinical attribute
hops	Hazardous or Poisonous Substance
bpoc	Body Part, Organ, or Organ Component
tisu	Tissue

Supplementary References

Aronhime J, Lifshitz-Liron R. (2009) Zoledronic acid crystal forms, zoledronate sodium salt crystal forms, amorphous zoledronate sodium salt, and processes for their preparation. Google Patents.

Cox J, Cartwright T, Neubauer M, McCollum D, Sandbach J, Monticelli M, Boehm K, Ilegbodu D, Asmar L (2006) A Phase II study of gemcitabine plus zoledronic acid in subjects with Stage IV pancreatic cancer. In *ASCO Annual Meeting Proceedings*, Vol. 24, p 14002.

Gottlieb A, Stein GY, Oron Y, Ruppin E, Sharan R (2012) INDI: a computational framework for inferring drug interactions and their associated recommendations. *Mol Syst Biol* **8:** 592

Huang J, Niu C, Green CD, Yang L, Mei H, Han J-DJ (2013) Systematic prediction of pharmacodynamic drug-drug interactions through protein-protein-interaction network. *PLoS Comput Biol* **9:** e1002998

Koto K, Murata H, Kimura S, Horie N, Matsui T, Nishigaki Y, Ryu K, Sakabe T, Itoi M, Ashihara E (2010) Zoledronic acid inhibits proliferation of human fibrosarcoma cells with induction of apoptosis, and shows combined effects with other anticancer agents. *Oncol Rep* **24:** 233

Li P, Huang C, Fu Y, Wang J, Wu Z, Ru J, Zheng C, Guo Z, Chen X, Zhou W (2015) Large-scale exploration and analysis of drug combinations. *Bioinformatics* **31:** 2007-2016

Park K, Kim D, Ha S, Lee D (2015) Predicting Pharmacodynamic Drug-Drug Interactions through Signaling Propagation Interference on Protein-Protein Interaction Networks. *PLoS One* **10:** e0140816

Su AI, Wiltshire T, Batalov S, Lapp H, Ching KA, Block D, Zhang J, Soden R, Hayakawa M, Kreiman G (2004) A gene atlas of the mouse and human protein-encoding transcriptomes. *Proc Natl Acad Sci U S A* **101:** 6062-6067

Tatonetti NP, Patrick PY, Daneshjou R, Altman RB (2012) Data-driven prediction of drug effects and interactions. *Sci Transl Med* **4:** 125ra131-125ra131

Trojan J, Kim S-Z, Engels K, Kriener S, Mitrou PS, Chow KU (2005) In vitro chemosensitivity to gemcitabine, oxaliplatin and zoledronic acid predicts treatment response in metastatic gastric cancer. *Anti-Cancer Drugs* **16:** 87-91

Zhao M, Tominaga Y, Ohuchida K, Mizumoto K, Cui L, Kozono S, Fujita H, Maeyama R, Toma H, Tanaka M (2012) Significance of combination therapy of zoledronic acid and gemcitabine on pancreatic cancer. *Cancer Sci* **103:** 58-66
